# Occurrence of type VI secretion system effector genes in longitudinal isolates of P. aeruginosa from people with cystic fibrosis

**DOI:** 10.1099/mgen.0.001555

**Published:** 2025-11-07

**Authors:** Antonia Habich, Ying Liu, Melanie Ghoul, Sandra B. Andersen, Helle Krogh Johansen, Søren Molin, Ashleigh S. Griffin, Daniel Unterweger

**Affiliations:** 1 Institute for Experimental Medicine, Kiel University, Michaelisstraße 5, 24105 Kiel, Germany; 2 Max Planck Institute for Evolutionary Biology, August-Thienemann-Straße 2, 24306 Plön, Germany; 3 Department of Biology, University of Oxford, Oxford, UK; 4 Center for Evolutionary Hologenomics, GLOBE Institute, University of Copenhagen, 1353 Copenhagen, Denmark; 5 Department of Clinical Microbiology 9301, Rigshospitalet, Copenhagen, Denmark; 6 Department of Clinical Medicine, Faculty of Health and Medical Sciences, University of Copenhagen, Copenhagen, Denmark; 7 The Novo Nordisk Foundation Center for Biosustainability, Technical University of Denmark, Lyngby, Denmark

**Keywords:** Comparative genomics, Pseudomonas aeruginosa, Type VI Secretion System

## Abstract

*Pseudomonas aeruginosa* uses multiple type VI secretion systems (T6SSs) to manipulate eukaryotic cells, kill competing microbes and take up nutrients. Bacterial strains are known to differ in their T6SS apparatus and the toxic effector proteins responsible for killing. The ability to eliminate competitors has been repeatedly demonstrated in lab studies, but much less is known about effector genotypes during infection. We used comparative genomics to test for the presence and absence of T6SS effector genes in over 450 clinical *P. aeruginosa* isolates from people with cystic fibrosis in Copenhagen (Denmark) and complemented these findings with data of 52 isolates from people with cystic fibrosis in London (UK). We found natural variation in the occurrence and combination of effector genes. Patients were typically infected with isolates that differ in their effector gene sets but show no statistically significant association between the number of effector genes and chronic infection. Isolates with a pair of T6SS effector and immunity genes and isolates without these genes, which would be expected to kill each other based on existing work in the laboratory, were found on the same individual. Taking the isolates’ phylogeny and sampling times into account, we identified five putative loss events of effector genes during infection. Although the impact of our findings for infected individuals will require further investigation, we demonstrate the extent of strain-level variation in T6SS effector genes in clinical isolates.

**Impact Statement**

*Pseudomonas aeruginosa* strains are known to carry a diversity of genes that encode proteins of the type VI secretion system (T6SS), a molecular machinery to kill competing microbes, manipulate eukaryotic cells and take up nutrients. However, we have limited information about how patterns of variation associate with different stages of infection or how alleles of the same gene vary between strains or between host infections. We take advantage of the opportunity to analyse patterns of variation in T6SS genes of *P. aeruginosa* isolates longitudinally sampled from people with cystic fibrosis. Our findings show variation in the occurrence of T6SS effector genes among clinical isolates. The implications for infection and for competition between strains within patients require further investigation.

## Data Summary

This study is based on publicly available genome data. Accession codes are provided in Tables S1 and S2 (available in the online Supplementary Material). All supplementary tables have been uploaded to the Figshare account of the Microbiology Society [1] (link: https://doi.org/10.6084/m9.figshare.30003217.v1). The authors confirm that all supporting data has been provided within the article or in the supplementary material.

## Introduction

*Pseudomonas aeruginosa* is an opportunistic pathogen that colonizes and chronically infects the lungs of people with cystic fibrosis (CF) [2]. These infections can decrease an individual’s lung function, impair their quality of life and become fatal [2,3]. Although bacterial infections are less of a burden for those patients who receive the new treatment with CF transmembrane conductance regulator modulators, *P. aeruginosa* is still found in their lungs [4,5]. The bacteria evolve during chronic infection and adapt to the host environment typically by adopting a biofilm-associated lifestyle and reduced virulence [6,7]. Individual patients are typically colonized by a single lineage of bacteria that diversifies during chronic infection [8]. Some individuals are infected with bacteria of more than one lineage during their medical history [9,10]. During colonization and throughout the infection, *P. aeruginosa* cells will be challenged through antagonistic interactions with competing species of microbes and with the host. However, the mode by which these interactions take place and the strategies *P. aeruginosa* adopts to manipulate others and defend themselves are poorly understood [11].

Type VI secretion systems (T6SSs) are likely to play a critical role in mediating the interaction between *P. aeruginosa* cells and their competitors and host cells in the lung environment. The T6SS machinery consists of a contractile injection system that translocates diverse effector proteins into neighbouring prokaryotic cells, the extracellular space and eukaryotic cells [12], including cells that are highly relevant to infection. Up to four distinct T6SSs enable *P. aeruginosa* to kill other microbes, manipulate eukaryotic cells and take up nutrients (H1-, H2-, H3- and H4-T6SS) [13,18]. Whereas the H1-, H2- and H3-T6SS are found at a high occurrence across strains of the species, the H4-T6SS is rare and found in only about 1% of the strains [18]. The regulation differs between the individual systems and between strains [13,19,21]. Two recent studies highlight the potential role of T6SS-mediated microbe–host and microbe–microbe interactions during *P. aeruginosa* infections. The first used human organoids to demonstrate T6SS-dependent invasion of goblet cells by *P. aeruginosa*. The second analysed the airway microbiome of individuals infected with *P. aeruginosa* and suggests a reduced microbial diversity upon T6SS-mediated bacterial killing [22]. These findings raise interest in the T6SS genes of clinical *P. aeruginosa* isolates.

*P. aeruginosa* strains show natural variation in some of their T6SS effector genes. Genotypes of a strain with and without an effector gene, which are routinely generated in the laboratory by genetic engineering to study the T6SS, also do exist in nature [18]. We therefore expect that a strain’s phenotype observed in the laboratory, which is caused by the presence or absence of a T6SS effector, is relevant for bacteria in their natural environment and for pathogenesis during infection of a host. For example, rats that were infected with a laboratory reference strain with the H2-T6SS effector gene *pldA* succumbed faster to a *P. aeruginosa* infection than animals that were infected with a genetically engineered version of this strain without *pldA* [23]. The naturally occurring presence and absence of *pldA* among clinical isolates could similarly affect the disease course of infected individuals, as previously proposed for patients with acute and chronic infections [24]. Knowing about the occurrence of individual effector genes might therefore help to explain intraspecific differences in pathogenicity. Additionally, bacteria–bacteria killing by PldA was observed in the laboratory when bringing a strain with the effector (*pldA*) and immunity gene (*tli5a*) in direct contact with a genetically engineered strain that lacks these genes [25]. In a patient, such T6SS-mediated interactions could happen between co-occurring strains that naturally differ in their effector sets. Knowing about the occurrence of individual effector genes might help to understand bacteria–bacteria interactions during infection. So far, little is known about the presence and absence of T6SS effector genes in clinical isolates from people with CF across cohorts and about T6SS-mediated phenotypes in infected patients, which are expected based on laboratory experiments.

**Fig. 1.**
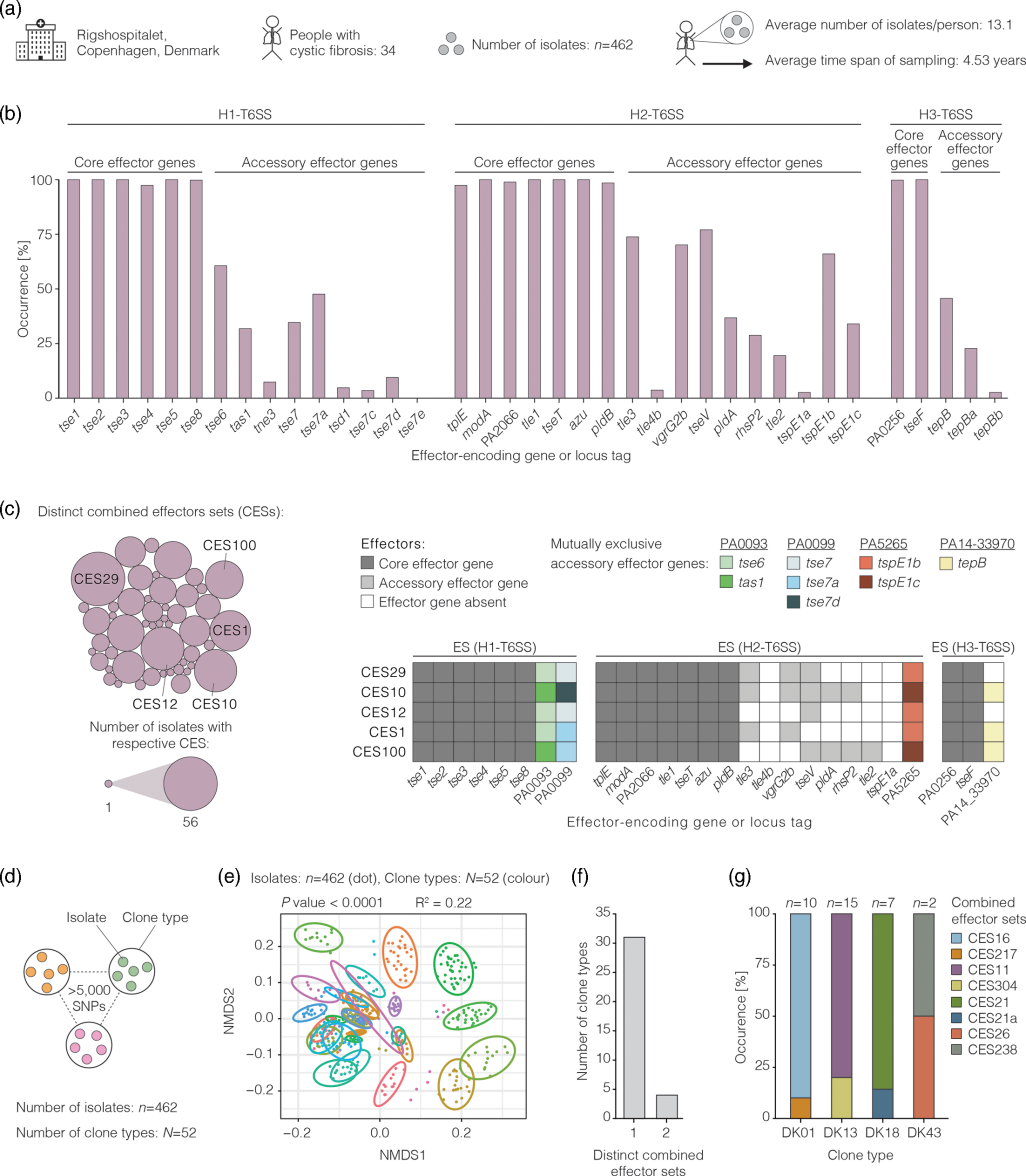
Isolates of patients from Copenhagen differ in the presence and absence of multiple T6SS effector genes. (a) Overview of the existing isolate collection that was first presented by Marvig and colleagues [9]. (b) Bar graph indicating the occurrence of core and accessory effector genes. (c) Bubble graph shows the distribution of distinct combined effector sets (CESs) across the isolate collection from Copenhagen. Each bubble indicates one distinct CES. The size of the bubble indicates the number of isolates with the respective CES. The five most frequently observed effector sets are shown on the right. (d) The isolates of the collection were previously grouped into 52 clone types [9,54]. Isolates of different clone types differ by at least 5,000 SNPs. (e) Separation of the isolates by their T6SS effector genes. Non-metric multidimensional scaling (NMDS) plot based on a Jaccard distance matrix. Each dot represents one genome (*n*=462). Dots and ellipses are coloured by clone type (extended figure with colour legend is provided in Fig. S7). Statistical significance was calculated with a permutational multivariate analysis of variance test with 10,000 permutations. (f) Number of clone types with all isolates of a clone type encoding the same set of T6SS effector genes or encoding distinct CESs. Included are only clone types with at least two isolates in the collection. (g) Occurrence of a CES among isolates of a clone type. Only clone types are shown that have isolates with more than one CES. The respective CESs are depicted in Fig. S8.

Here, we performed comparative genomics on T6SS effector genes of *P. aeruginosa* isolates from lung infections of patients with CF. The genomes belong to an existing collection of 462 isolates that were collected longitudinally from 34 patients in Copenhagen since the onset of the infection (Fig. 1). Multiple existing studies on this collection identified genome-wide patterns of bacterial adaptation to the CF lung [9,26,33]. We specifically focused on the three most common T6SSs and investigated the presence and absence of their T6SS effector genes among isolates of each individual over the timespan of sampling. Our analyses revealed the following observations: (i) evidence to suggest that patients are typically initially colonized with isolates with different sets of effector genes, (ii) no evidence that the number of effector genes influences ability to establish a chronic infection, (iii) *P. aeruginosa* isolates sampled from the same patient at the same timepoint showed naturally occurring differences in T6SS effector sets that were reported to cause T6SS-mediated killing between genetically engineered strains in laboratory experiments, (iv) five examples of T6SS effector gene loss during chronic infection in three patients, (v) consistent occurrence patterns of T6SS effector genes across patient cohorts of two geographic locations. Our work demonstrates natural variation in T6SS effector genes as one of many genomic differences between clinical isolates of CF patients.

## Methods

### Isolate collection and cohort

This study is based on existing isolate collections from Copenhagen [9] and London [34]. The isolates originate from individuals with a CF diagnosis at the University Hospital, Rigshospitalet, Copenhagen, Denmark, and the Royal Brompton Hospital, London, UK. For the individuals of the Copenhagen cohort, the median age is 8.8 years at the timepoint of the first sequenced *P. aeruginosa* isolate [9]. Data on the timepoints of sampling is provided in the original studies in Table S7 (Copenhagen isolates [9]) and Table S1 (London isolates [34]). Details on the antibiotic treatment of individuals of the cohort from Copenhagen are provided in Table S8 of the original study [9]. The sampling from sputum is described in detail in the original studies [9,34]. Few isolates from individuals in London were isolated not from sputum but from cough swabs (PALA5, PALA27, PALA28, PALA29, PALA33, PALA34, PALA35, PALA37 and PALA44).

### Accession of genomes

Existing genome assemblies of the isolate collection from Copenhagen were downloaded from ENA on 5 May 2021, and genome assemblies of the London isolate collection were downloaded from the NCBI on 12 January 2023. Accession codes for the isolate collection from Copenhagen can be found in Table S1. Accession codes for the isolate collection from London can be found in Table S2. We assessed the quality of the assembled genomes using QUAST [35,36] (version 5.0.0) and report among others on N50 values, L75 values and %GC content (Tables S20 and S21). Eleven genomes of the isolate collection from Copenhagen (CPH_136, CPH_136_1, CPH_137, CPH_138, CPH_140, CPH_140_1, CPH_141, CPH_142, CPH_143, CPH_146 and CPH_147) were excluded from this analysis because of insufficient quality at genomic loci relevant to T6SS genes. The study was performed on a total of 462 genomes of the Copenhagen collection.

### Occurrence analysis of T6SS effector genes

The occurrence of effector genes in the datasets of assembled genomes from Copenhagen and London was performed as described in Habich *et al*. [18]. In short, we extracted nt sequences encoding for known *P. aeruginosa* T6SS genes from reference strains PAO1 (NC002516), PA14 (NC_008463.1) and PAK (NZ_CP020659.1) and from the clinical strains PALA36 (CP110348) and PALA52 (CP110347) (Table S4). These sequences were used as queries for a local blastn search. The blastn search and calculation of nt identity were performed in the same way as described in the section on the Occurrence analysis of T6SS apparatus genes (see below). A hit of >80% was considered positive for effector gene presence. The absence of an effector gene was confirmed by including neighbouring genes into the analysis. For inconclusive hits, genomes were manually analysed in Geneious (version 2019.2.3).

### Identification of orphan immunity genes

To test for orphan immunity genes, we performed a comparative analysis. nt sequences of known immunity genes were extracted from the genomes of reference strains and clinical strains (Table S4). These sequences were used as queries in a local nucleotide-nucleotide blast analysis against the assembled genomes of the Copenhagen dataset as described for the occurrence of effector genes. Hits with an nt identity of at least 80% and an alignment length of at least 90% were considered positive. We then tested if the cognate effector gene was encoded in the same genome based on the existing results of the occurrence analysis of the effectors. Immunity genes without a cognate effector gene encoded in the same genome were considered orphan immunity genes.

### Occurrence analysis of T6SS apparatus genes

To test for the presence or absence of the three previously reported T6SS apparatus gene clusters in the dataset from Copenhagen, we used a previously established workflow [18]. In short, we performed a local blastn search using individual genes from *P. aeruginosa* reference strain PAO1 (H1-T6SS: PA0074-91, H2-T6SS: PA1656-71, H3-T6SS: 2359-73, PAO1 accession number: NC_002516, Table S3) as queries and the dataset of assembled genomes as a database. We used a nucleotide-nucleotide blast (version 2.16.0+) with default settings and output format 10. As described in detail in Rohwer *et al*. [37], we then calculated the nt identity score of the blast hits to the query with the following equation: nt identity = (pident*length)/(qlen+(length-(qend-qstart+1))) with pident, length, qlen, qend and qstart being output values from the blast search. A blast hit with at least 95% nt identity was considered a positive hit. Genomes with positive hits for at least 12 apparatus genes were considered to encode the respective T6SS. The H4-T6SS apparatus gene cluster was excluded from the study because the effectors of this system are currently unknown.

To additionally test for a functional T6SS in isolates that lost a T6SS effector gene, we extracted the nt sequence of the genomic region of the respective T6SS apparatus gene cluster (H1-T6SS cluster for CPH_421 and H2-T6SS cluster for CPH_120, CPH_121, CPH_114 and CPH_412_1). These sequences were aligned to the respective genomic region in PAO1. nt alignments are reported in Fig. S12. Additionally, we analysed T6SS apparatus genes one by one for frameshift mutations and mutations leading to premature stop genes.

### Comparison of T6SS regulatory genes

Sequencing reads were mapped to the *P. aeruginosa* PAO1 reference genome using breseq to assess variation in T6SS regulatory genes across strains and longitudinal isolates. Reads were trimmed and quality-checked using fastp, FastQC and MultiQC. Breseq results were manually inspected to identify non-synonymous mutations, insertions and deletions in regulatory genes that could affect T6SS functionality.

### Non-metric multidimensional scaling analysis

The separation of isolates of different clone types by their combined effector sets (CESs) was tested with a non-metric multidimensional scaling (NMDS) analysis using the R package vegan [38]. We computed a Jaccard distance matrix based on the function avgdist() and the option dmethod=‘jaccard’. The actual NMDS analysis was performed with the metaMDS() function. To statistically test if the CESs of the isolates correlate with their respective clone types, we used the function adonis2() with 10,000 permutations and controlled for patient origin. Results were plotted with the R package ggplot2 [39].

### Phylogenetic analysis of genomes

Phylogenetic trees were built on a core genome alignment and generated in multiple steps. First, the genomes were annotated with Prokka [40] (version 1.14.5). Second, we identified the core genome by performing a pan-genome analysis with Panaroo [41] (version 1.3.2). We used default settings and mode ‘moderate’. Option ‘-a core’ was chosen to build a concatenated core genome alignment based on genes with an occurrence of at least 99%. This core genome alignment (output file ‘core_gene_alignment_filtered.aln’) was then used to compute the maximum-likelihood phylogenetic tree. The tree was inferred using IQ-TREE [42,43] (version 1.6.12) with ModelFinder [44] (option ‘-m MFP’) to identify the best fit model based on the Bayesian information criterion (BIC) and with 1,000 ultrafast bootstrap replicates (option ‘-bb 1000’). Due to computational limits, the model for trees with more than 400 genomes was not chosen based on the core genome alignment. Instead, we randomly selected ten genomes for each core gene and built multiple sequence alignments using muscle [45] (version v3.8.31). Then, the best-fit model was chosen for each of those alignments separately based on the BIC using ModelFinder. We enumerated how often a model was chosen. The model that was considered most often was used to compute the phylogenetic tree. The tree was then inferred from the core genome alignment with 1,000 ultrafast bootstrap replicates. The model used to infer a particular tree is indicated in the figure legend. Trees were rooted as indicated in the figure legends and plotted using the R package ggtree [46].

### Alignments and read mapping

nt alignments of two sequences were performed in Geneious (version 2019.2.3) using muscle [45] (version v3.8.425). Reads were extracted from the NCBI between January and March 2025 and mapped to the reference strain PAO1 in Geneious using the ‘map to reference’ function.

### Identification of mutations in isolates of the same clone type

The identification of mutations between two isolates of clone type DK01, CPH_433 and CPH_421, was performed in two steps. First, isolate CPH_433 was *de novo* assembled. Second, reads of isolate CPH_421 were mapped to the *de novo* assembled genome of CPH_433. The raw reads of both isolates were downloaded from the NCBI. Adapter trimming was performed with fastp [47] and the quality control with FastQC [48] and MultiQC [49]. The trimmed reads from isolate CPH_433 were *de novo* assembled with Unicycler [50] followed by quality control with QUAST [35,36] (version 5.0.0). The scaffolds were assembled with RagTag [51] using the genome of PAO1 as a reference. Prokka [40] was used for genome annotation with the option –proteins to first annotate from the PAO1 reference genome. The trimmed reads from CPH_421 were mapped to the *de novo* assembled genome of CPH_433 using breseq [52].

### Analysis of effector genes by colonization and infection of people with CF

The first isolates of each person with CF (*n*=34) were grouped based on their colonization and infection pattern (Table S11): Isolates for which there were only isolates of the same clone type were considered strong infectors. Isolates for which there were isolates of the same but also of other clone types were considered weak infectors, and isolates for which there were only isolates of other clone types were considered colonizers. Unequal length of sampling time could introduce errors. For example, isolates of individuals with a short sampling time span could wrongly be classified into a strong infector. We therefore only considered individuals with a sampling time span of at least 1,000 days (Table S12). For each genome, the total number of effectors and the total number of effectors associated with one of the T6SSs were counted and compared between the groups. Statistical significance was calculated using a Kruskal–Wallis test in GraphPad Prism (version 10.4.1, for MacOS). Occurrences of single effector-encoding genes were also enumerated. Results were plotted in GraphPad Prism and statistical significance was tested with a chi-square test.

### Comparison of T6SS effector genes across isolate collections

The occurrence of effector genes in the two isolate collections from Copenhagen and London was plotted with the R package ggplot2 [39]. To test for significant differences in occurrence, Fisher’s exact test was used. *P* values were corrected for multiple testing [false discovery rate (FDR)=0.05] using the p.adjust() function of the R package stats [53]. *P* values below 0.05 were considered significant.

### Test for location-specific differences in effector sets

We performed random sampling using a customized R script. To avoid pseudo-replication, we included only one isolate per clone type. To choose these isolates of the collection from Copenhagen, we made use of the existing classification into clone types (*n*=52, Table S22). To choose these isolates of the London collection, we applied a cutoff at a distance of 0.000231 substitutions per site (largest distances of two isolates of the same clone type in the Copenhagen collection) to consider two isolates of the same clone type. One isolate per clone type was included for further analysis (*n*=36, Fig. S19). Effector sets of the remaining isolates from Copenhagen (*n*=52) and from London (*n*=36) were combined. Then, we randomly split the effector sets into two groups (one group with *n*=52 and the other with *n*=36) and counted the number of effector sets found in both groups. The random sampling step was performed 10,000 times and the *P* value was calculated with the following: # of same or less same effector sets/number of iterations.

### Identification of large genomic gaps

To identify the genomic gap that includes the effector gene PA2066, we combined read mapping and nt alignments when possible. First, we mapped raw reads of isolates lacking PA2066 (CPH_120, CPH_121 and CPH_114 of DK13 and CPH_412_1 of DK18) to PAO1 using breseq (version 0.39.0). To identify the start and end of the genomic gaps, we manually extracted the necessary information from the breseq output file ‘index.htlm’. We defined the genomic gap as the lack of continuous read mapping for at least 1,000 bp with a coverage of at least 50. Coverage plots of the genomic region of interest were generated using the BAM2COV function from breseq. To compare the genomic region additionally to the first isolate of the respective patient, we mapped the reads from the first isolate of the respective patient (CPH_122 for PID83337 and CPH_414 for PID80224) to PAO1. In this way, we were able to indirectly compare the genomic regions of interest between the first isolate and the isolates lacking the effector gene PA2066. Coverage plots were generated using the BAM2COV function from breseq. For CPH_120, we additionally performed a whole-genome alignment of the region of interest (here: from position 2,200,685 to 2,279,794 in the PAO1 reference genome) to PAO1 and also to the first isolate of the patient (CPH_122) using the MAUVE algorithm in Geneious.

### Graphical depictions

Illustrations for this manuscript were created with Adobe Illustrator (version 29.7.1).

## Results

### Occurrence of T6SS effector genes among clinical isolates from Copenhagen

To investigate the occurrence of T6SS effector genes in a collection of clinical isolates from one geographic location, we performed comparative genomics on *P. aeruginosa* isolates of people with CF followed at the CF clinic at Rigshospitalet in Copenhagen, Denmark. Our analysis is based on a customized database of T6SS effector genes that have been identified and characterized to date and originate from laboratory reference strains and clinical isolates (Tables S3 and S4). We focused on effector genes of the H1-, H2- and H3-T6SSs. The apparatus gene clusters of these three systems are present in all herein analysed isolates (Table S5). Across the three systems, 15 effector genes were found in at least 97% of the isolates (Figs 1b and S1–S3, Table S6), supporting their previous classification as core effector genes based on diverse strains across the species [18]. Twenty-one effector genes were found in 3 to 77% of the isolates, supporting their classification as accessory effector genes. Eleven isolates lack the effector gene *tse4* but do encode the corresponding immunity gene *tsi4*, showing rare cases of orphan immunity genes (Fig. S4). We found 50 distinct combinations of effector genes across the genomes (Figs 1c, S5 and S6, Table S4). Except for 2 out of 34 patients, each patient is colonized with isolates with different sets of T6SS effector genes. These findings demonstrate intraspecific diversity in the occurrence and in the combination of T6SS effector genes among patient isolates from Copenhagen.

The isolates of this collection have previously been grouped into clone types based on the overall similarity of their genomes [9,54] (Fig. 1d). Accordingly, isolates that differ by more than 5,000 SNPs in the core genome are considered a different clone type [54]. It is known that lineages of *P. aeruginosa* acquired and lost T6SS effector genes during their evolutionary history [18]. We therefore hypothesized that isolates of different clone types would be more likely to differ in their set of T6SS effector genes than isolates of the same clone type. Indeed, we observed a statistically significant association between the separation of the genomes by T6SS effector genes and by clone type (permutational multivariate analysis of variance, number of permutations=10,000, *P*<0.0001*,* $R^{2}$= 0.497; Figs 1e and S7). Genomes of the same clone type encoded the same T6SS effector genes except for isolates of the clone types DK01, DK13, DK18 and DK43 (Figs 1f, g and S8).

Together, the herein observed diversity in T6SS effector gene occurrence raises questions about (i) the role of the T6SSs for chronic infection, (ii) the co-occurrence of isolates with differing effector sets as a pre-requisite for intraspecific killing in the patient, (iii) changes in effector sets during infection and (iv) consistency across isolate collections. We will address these questions in the following sections by focusing exclusively on the herein observed occurrence of effector genes. We acknowledge that there is much more genomic variation among these isolates beyond T6SS effector genes, including slight variation in the nucleotide sequences of T6SS regulatory genes with unknown consequences on T6SS activity (Tables S7–S10).

### No indication for the impact of T6SS effector diversity on infection history

**Fig. 2.**
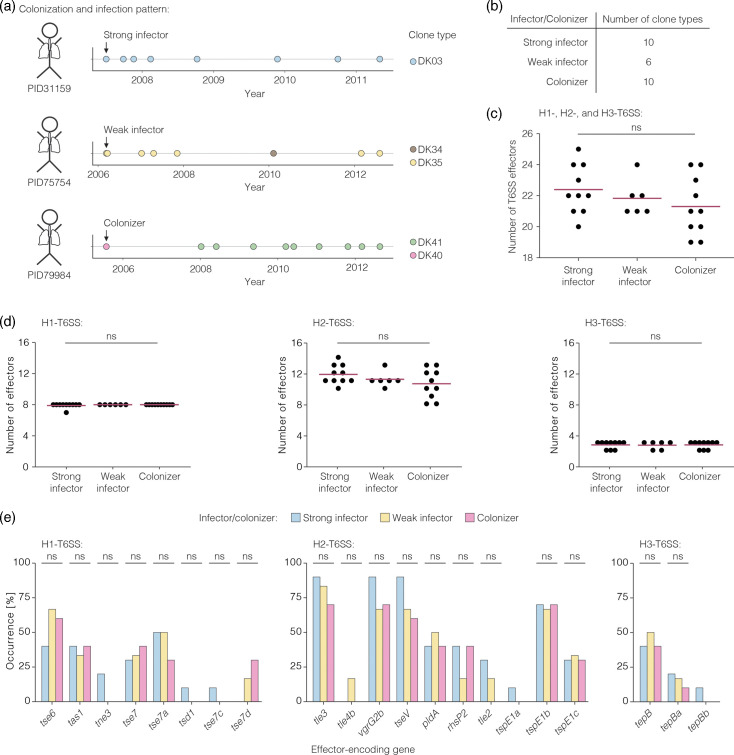
No association between the number of T6SS effector genes and infection. (a) Exemplary patterns of the initial colonization and subsequent infection of three individuals by *P. aeruginosa* isolates. Individuals were regularly monitored for *P. aeruginosa,* and the isolate that is highlighted with the arrow is from the first timepoint at which *P. aeruginosa* was detected in these individuals. (b) Table indicating the number of individuals per colonization and infection pattern. Only individuals with a sampling time of more than 1,000 days were considered for this analysis. (c, d) Association between a clone type’s number of effector genes and its ability to establish a stable infection in individuals. We assessed an association for the effector genes of the H1-, H2- and H3-T6SS altogether (**c**) and separately per T6SS (**d**). Each dot represents one clone type. The red line indicates the mean. Statistical significance was calculated using the Kruskal–Wallis test. (e) Occurrence of accessory effectors of the three T6SSs in clone types categorized as strong infector, weak infector or colonizer. Statistical significance was calculated with a chi-square test. Detailed results can be found in Table S14.

Laboratory experiments have shown that effectors of the H1-, H2- and H3-T6SS mediate an advantage to *P. aeruginosa in vitro* and *in vivo* [14]. We therefore hypothesized that T6SS effector genes facilitate the initial colonization and subsequent infection of an individual and tested an association between a clone type’s effector genes and its infection trajectory. Two pre-requisites for such an analysis were fulfilled: (i) the observed diversity in T6SS effector genes between clone types and (ii) differences between clone types in their frequency of detection during the timespan of sampling. We subdivided the clone types into three groups based on the initial colonization and infection of individuals (Fig. 2a, Table S11): first, clone types that were detected at the initial timepoint of sampling and exclusively found at sampling events thereafter (‘strong infectors’); second, clone types that were present at the initial sampling, absent at subsequent sampling events and present again at later timepoints (‘weak infectors’); and third, clone types that were found at the initial sampling only and not detected any more at later timepoints (‘colonizers’). To account for an error introduced by the unequal length of sampling time across individuals, we only included individuals that were sampled for more than 1,000 days (Fig. S9). This subgrouping resulted in ten strong infection clone types, six weak infecting clone types and ten colonizing clone types (Fig. 2b, Table S12).

First, we tested for differences in the overall number of T6SS effector genes between strong infectors, weak infectors and colonizers. We found that strong infectors had a mean number of 22.4 effector genes, whereas colonizers had a mean number of 21.3 effector genes (Fig. 2c). This difference was not statistically significant (Kruskal–Wallis test, *P*=0.41, Table S13). A previous report had noted more intraspecific variation in the number of effector genes of the H2-T6SS compared to the H1- and H3-T6SS [18]. We therefore separately tested the number of effector genes of either the H1-, H2- or H3-T6SS for an association to the clone types’ initial colonization and infection of individuals. We found no differences in the number of H1- or H3-T6SS effector genes and only minor differences in the number of H2-T6SS effector genes (Fig. 2d, Table S13). Strong infectors had a mean number of 11.8 H2-T6SS effector genes and colonizers had 10.6. Again, these differences were not statistically significant (Kruskal–Wallis test, *P*=0.39).

Second, we tested whether accessory effector genes were associated with strong or weak infectors or colonizers. Therefore, we analysed the occurrence of each accessory effector gene among the three different groups (Fig. 2e). Some accessory effector genes like *tse7a, tle3, vgrG2b* and *tseV* were present with a slightly higher occurrence in strong infectors than in weak infectors or colonizers. Other effector genes like *tse6* or *pldA* were found with a slightly lower occurrence in strong infectors compared to weak infectors or colonizers. However, we did not find statistically significant differences (chi-square test, *tse6 P*=0.52, *pldA P*=0.91, full list in Table S14). Taken together, we could not explain the differences between clone types in their infection trajectory with differences in their T6SS effector genes, which might not be surprising given the overall low occurrence of some of the effector genes, the multiple factors that contribute to an infection and the antibiotic treatment of the individuals.

### Diversity in effector sets as a pre-requisite for T6SS-mediated competition in the patient

**Fig. 3.**
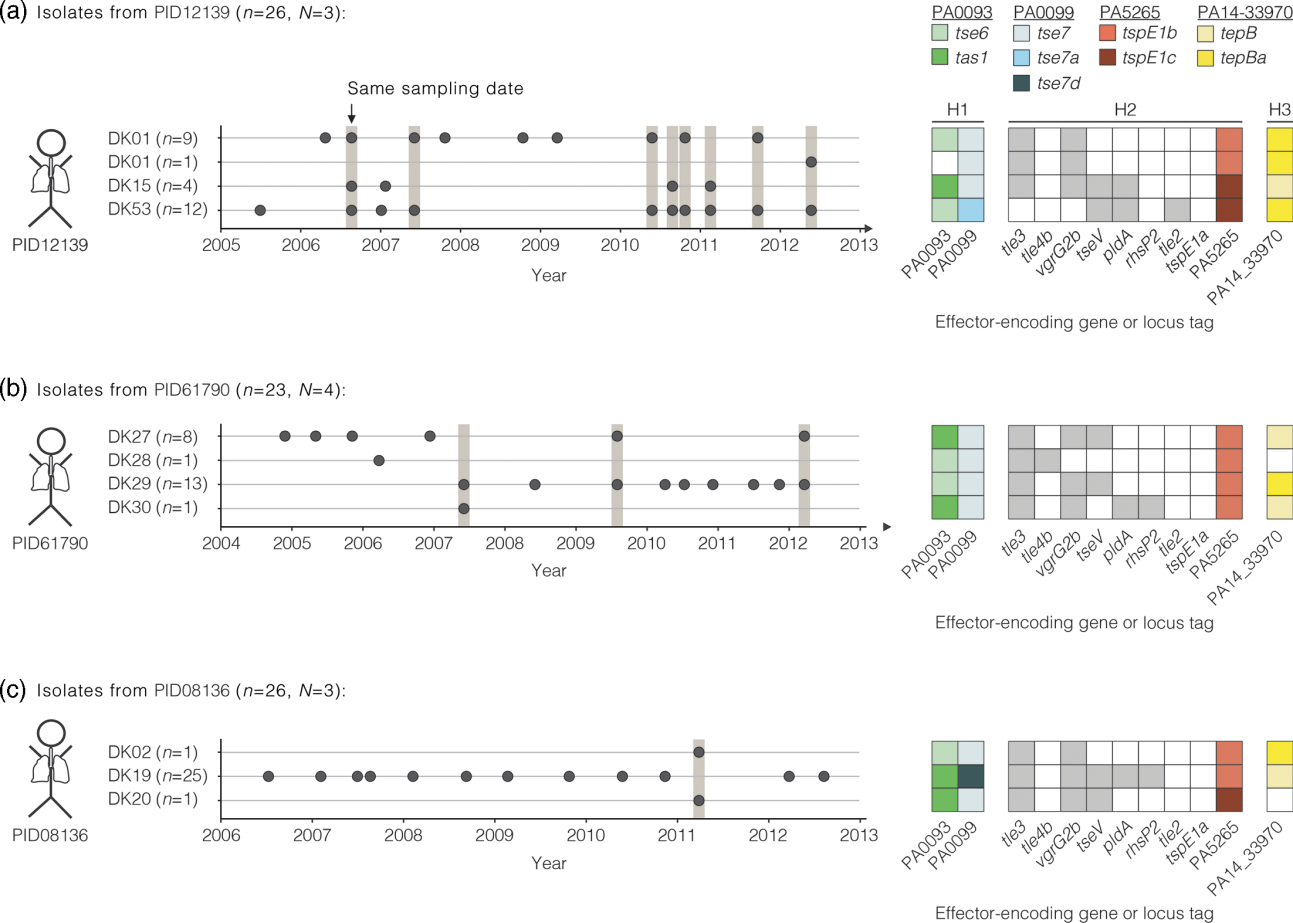
Isolates with differing effector sets were collected at the same sampling date. Longitudinal sampling of the isolates from the individuals PID12139 (**a**), PID61790 (**b**) and PID08136 (**c**). Each dot refers to an isolate and the timepoint at which it was collected. Shading indicates the sampling of multiple isolates at the same sampling date. The accessory effector genes of the respective isolates are shown on the right. The presence (filled box) and absence (white box) of genes is indicated.

Strains with differing T6SS effector sets have been mixed in laboratory experiments and shown T6SS-mediated bacterial killing [55]. It is so far unknown whether such strains encounter each other during infection, which would be a pre-requisite for T6SS-mediated killing between *P. aeruginosa* strains in a patient. We tested for differences in the effector gene repertoires between isolates from the same patient as a proxy for isolates that could encounter each other during infection. In all three patients with multiple isolates from the same sampling date, we found differing effector gene repertoires among diverse clone types (Fig. 3). One individual is PID12139, who is infected by isolates of the clone types DK01, DK15 and DK53. At multiple dates, isolates of at least two clone types were simultaneously isolated from sputum samples. Isolates vary between the three clone types in two effector genes of the H1-T6SS, six effector genes of the H2-T6SS and one effector gene of the H3-T6SS (Fig. 3a). Another individual is PID61790, who is infected by isolates of clone types DK27, DK28, DK29 and DK30. Isolates of these clone types vary in seven effector genes of all three T6SSs (Fig. 3b). Yet another individual is PID08136, who is infected by isolates of clone types DK02, DK19 and DK20 (Fig. 3c). All of these isolates that lack a particular effector gene at a given genomic locus also lack the respective immunity protein-encoding gene, excluding the possibility of orphan immunity genes that would protect strains from being killed without encoding the effector gene itself. It remains an open question whether these isolates from the same individual actually got in direct contact or are spatially separated as observed in studies on lung explants [8]. We also found these differences in effector genes between isolates from the same individual and different sampling timepoints (Fig. S10). It remains unknown if T6SS-mediated killing between *P. aeruginosa* strains occurs in a patient. So far, we are not able to explain the infection trajectory of patients with potential T6SS-mediated killing between co-occurring clone types. However, our findings show that isolates with differing T6SS effector gene repertoires can be collected from the same patient, so such interactions could occur.

### Putative loss of effector genes in individuals during chronic infection

**Fig. 4.**
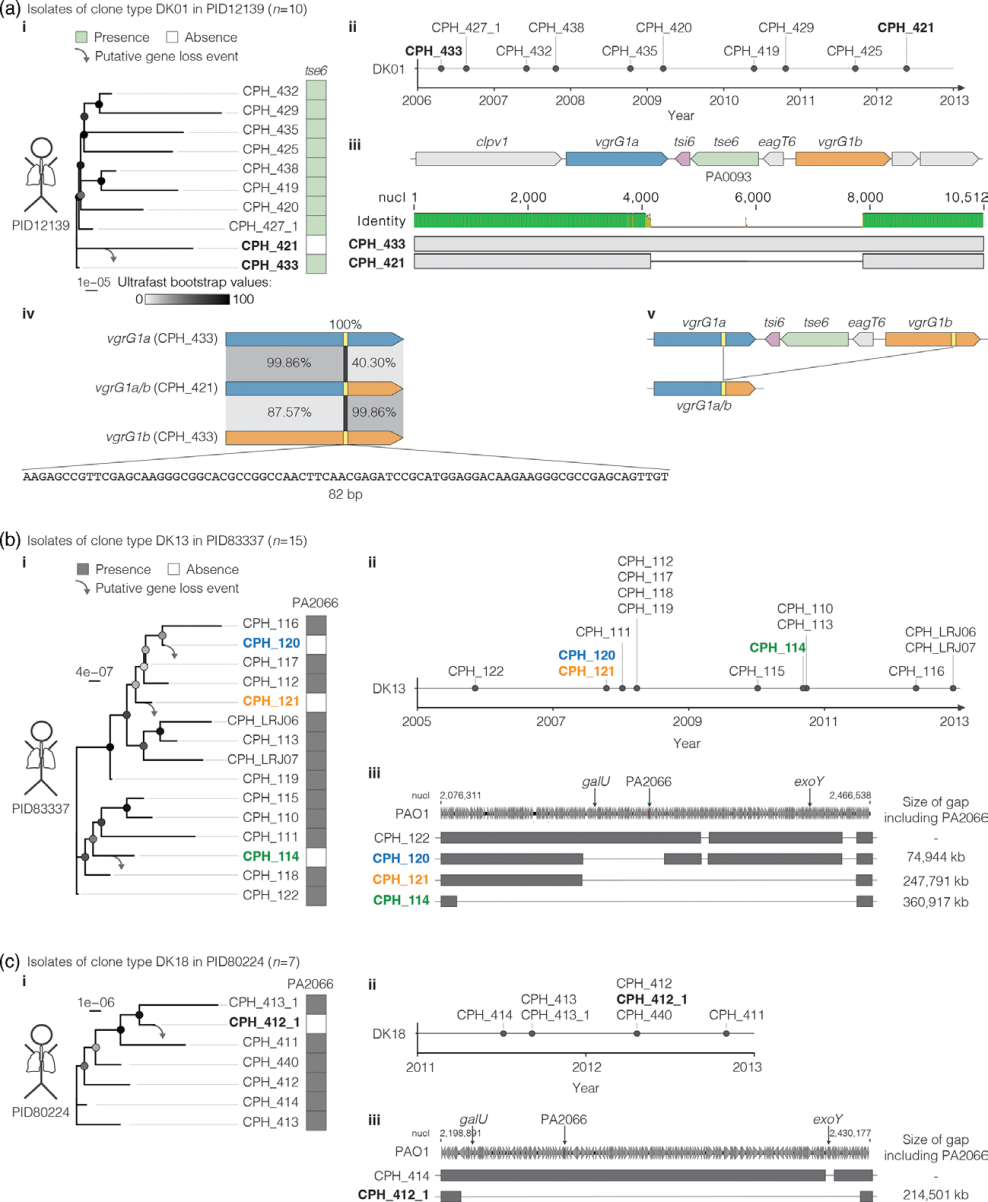
Closely related isolates of a few individuals differ in the presence and absence of T6SS effector genes. (a) Presence and absence of the effector gene *tse6* among isolates of the clone type DK01 from individual PID12139. (i) Phylogenetic tree based on a core genome alignment. The maximum-likelihood tree was inferred with the HKY+F+I model and is rooted to the isolate with the earliest sampling date (CPH_433). Presence (green) or absence (white) of the effector gene *tse6* is indicated. (ii) Depiction of the timepoints at which the isolates were collected from this individual. (iii) nt alignment of a genomic region that spans the effector gene *tse6* and neighbouring T6SS genes *tsi6*, *eagT6*, *vgrG1a* and *vgrG1b* of isolates CPH_433 and CPH_421. (iv) Schematic of the nucleotide alignment of *vgrG1a* and *vgrG1b* of isolate CPH_433 and of *vgrG1a/b* of isolate CPH_421 with indicated nt similarities. (v) Schematic of a putative recombination event within the *vgrG* genes that results in an in-frame deletion and the loss of *tse6*. (b) Presence and absence of the effector gene PA2066 among isolates of the individual PID83337. (i) Phylogenetic tree based on a core genome alignment. The maximum-likelihood tree was inferred with the HKY+F+I model and is rooted to the isolate with the earliest sampling date (CPH_122). Presence (grey) or absence (white) of the effector gene PA2066 is indicated. (ii) Timeline with the sampling dates of the individual isolates. (iii) Depiction of large gaps of varying length in each of the three isolates that lack the effector gene PA2066. The genomic region of PAO1 is indicated as a reference. Grey boxes indicate an average coverage of at least 50. (c) Presence and absence of the effector gene PA2066 among isolates of the individual PID80224. (i) Maximum-likelihood tree based on a core genome alignment with inference of the HKY+F+I model. The tree is rooted to the isolate with the earliest sampling date (CPH_414). (ii) Timeline of the sampling from this individual. (iii) Depiction of a large gap in isolate CPH_412_1 that includes the effector gene PA2066. Phylogenetic trees with exact bootstrap values are shown in Fig. S17.

T6SS effector genes are encoded on mobile genetic elements and were frequently gained and lost during the diversification of the species [18]. We hypothesized that a strain’s repertoire of effector genes also changes during chronic infection of individuals. We therefore combined the data on effector gene occurrence with core-genome-based phylogenies and data on the timepoints of sampling. We focused on isolates of the same individual and of the same clone type to specifically test for genomic changes that likely occurred during chronic infection (Fig. S11). We consider these longitudinal isolates as genetically related members of a bacterial population within a patient. In 3 out of 34 individuals, we found indications for the loss of the effector genes *tse6* and PA2066 during chronic infection (Fig. 4). These cases are described in detail in the following paragraphs.

The effector gene *tse6* was absent from one out of ten isolates of clone type DK01 in individual PID12139 [Fig. 4a(i, ii)], suggesting one loss event. Tse6 is an experimentally characterized NAD(P)+ glycohydrolase that mediates bacterial killing and is secreted by the H1-T6SS apparatus [56]. Analysis of the apparatus genes suggests that the T6SS itself remains functional after the loss of the effector protein (Fig. S12a). To gain insight into the mechanism by which *tse6* might have been lost, we aligned the genomic locus of the isolate without *tse6* (CPH_421) to the closest relative isolate with *tse6* (CPH_433). Isolate CPH_421 lacks 4,332 bp, which includes the genes *tse6*, *tsi6* and *eagT6* and parts of the adjacent *vgrG* genes [Figs 4a(iii) and S13, Table S15]. The proteins encoded in these genes comprise a functional unit consisting of the effector Tse6 and its adaptor protein EagT6, which enables the secretion of Tse6 by the VgrG protein [57]. The immunity protein Tsi6 inhibits the toxic activity of Tse6 and protects sister cells from getting killed [56]. A conserved sequence in the flanking *vgrG* genes, which are a known hotspot for recombination [18,58, 59], suggests the loss of *tse6* by an in-frame deletion and the generation of a new chimeric *vgrG1a/b* gene [Fig. 4a(iv, v)].

The effector gene PA2066 was absent from 3 out of 15 isolates of clone type DK13 of individual PID83337 and from 1 out of 7 isolates of clone type DK18 of individual PID80224 (Fig. 4b, c). The phylogenies based on the core genome alignment suggest four independent loss events of the gene as part of large genomic deletions of up to 280 kb (Figs S14–S17, Tables S16 and S17). We did not find an indication for mutations in apparatus genes of the H2-T6SS that would compromise T6SS functionality particularly in strains without the effector (Fig. S12b). The effector protein encoded in PA2066 is known to have antibacterial activity [60]. Among the many other genes that are absent together with PA2066 are *galU* and *exoY*. GalU is a uridylyltransferase and its loss has previously been described to result in a modification of lipopolysaccharide [61]. In infection experiments with mice, a mutant of *P. aeruginosa* PAO1 that lacks *galU* showed lower viable counts in the lung than the WT strain [62]. ExoY is an effector protein of the type III secretion system and a known virulence factor of *P. aeruginosa* [63,64]. The physiological relevance of the loss of PA2066 alone therefore remains unclear. The isolates with the deletion are only detected sporadically and not picked up consecutively, which could be an indication of reduced fitness after loss of this genomic segment.

Taken together, we observed multiple examples of putative effector gene loss during chronic infection. In the absence of population data of the individual timepoints, we are unable to measure bacterial fitness in patients after gene loss, which could be increased, decreased or remain unchanged. More broadly, our findings highlight dynamic changes in the T6SS effector gene repertoire within a few patients. The loss of effector genes is overall rare. No change in the effector gene repertoire was observed in 31 out of 34 patients, and no gene loss was observed for 35 out of 37 effector genes.

### Similar occurrence of T6SS effector genes across isolate collections sampled from patients across geographic regions

**Fig. 5.**
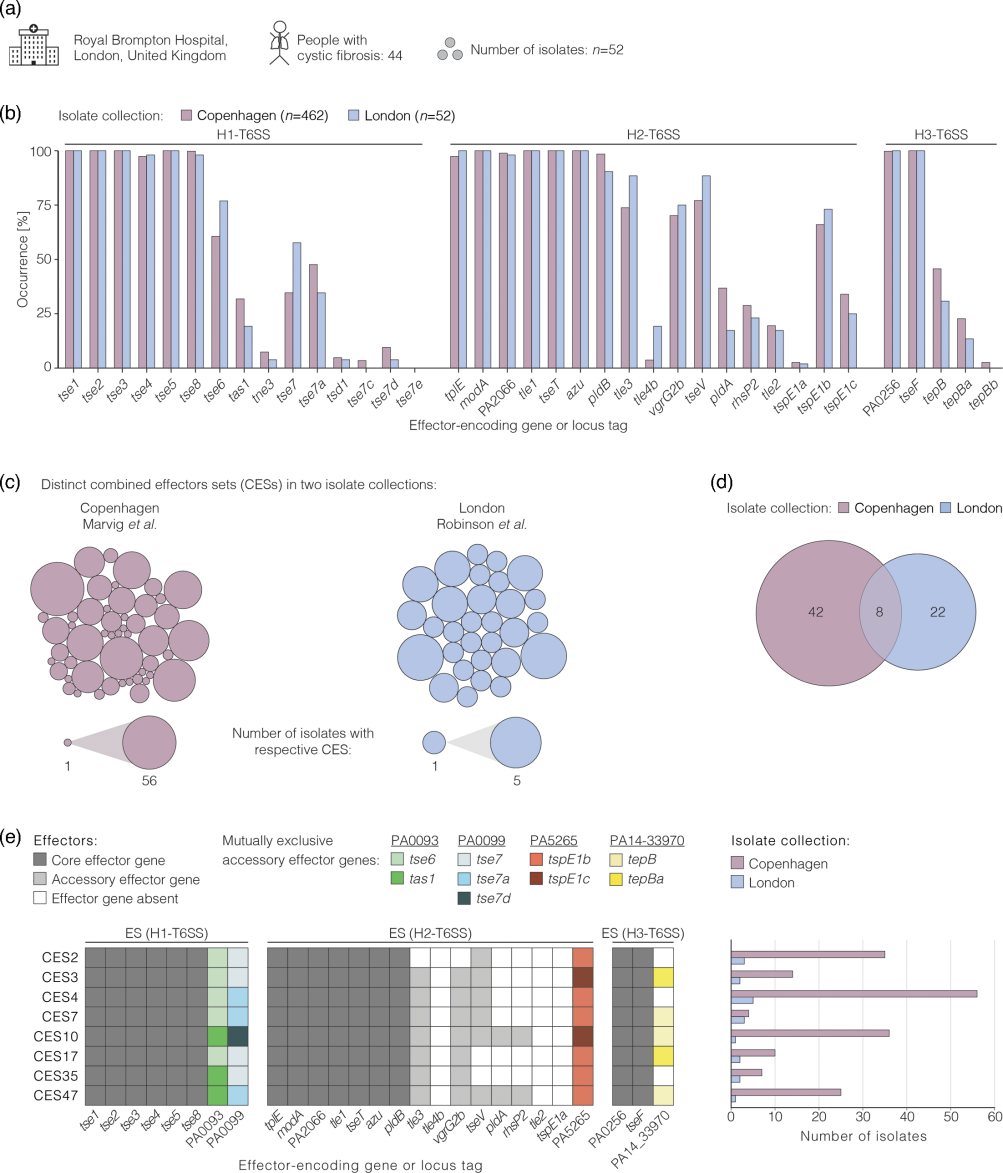
Similar occurrence of effector genes across isolate collections of individuals with the same underlying condition. (a) Overview of the isolate collection from that was previously studied by Robinson and colleagues [34]. (b) Bar graph shows the occurrence of the indicated effector genes in isolates of people with CF from Copenhagen (*n*=462) and London (*n*=52). (c) Bubble graphs indicate the distribution of distinct CESs across the genomes of the collections from Copenhagen and London. Each bubble indicates one distinct CES. The size of the bubble indicates the number of strains with the respective CES. (d) Venn diagram indicating the number of CESs that are found in either of the two isolate collections or in both. (e) Graphical depictions of the CESs that are found in both collections. Each box indicates the presence of an effector gene. Core effector genes are indicated in dark grey and accessory effector genes in light grey or in colour if mutually exclusive. The bar graph on the right indicates the number of strains with the respective CES in each collection.

Having observed the occurrence of T6SS effector genes among clinical isolates from Copenhagen, we hypothesized that effector genes also vary among isolates of patients with the same condition from another geographic location. To test this hypothesis, we compared the effector gene occurrence of the isolates from patients with CF in Copenhagen to *P. aeruginosa* isolates from patients with CF in London, UK [34] (Fig. 5a). The collection from London included isolates of the phylogroups A, B and C, as was the case for the isolates from Copenhagen, and their effector genes had previously been identified by Robinson *et al.* [34] (Table S18). No statistically significant difference was observed between the two collections in the occurrence of 36 out of 37 effector genes (Fisher’s exact test, FDR=0.05, list of *P* values for all genes in Table S19) (Fig. 5b). *Tle4b*, which encodes an effector with putative alpha/beta-hydrolase activity [34], has a 17% higher occurrence in the isolates from London (Fisher’s exact test, FDR=0.05, *P*=0.004). Further, we analysed the effector sets in isolates of the two locations. We found more than 20 different effector sets among isolates at each location, demonstrating a high diversity that could even be bigger when considering so far unknown effector genes that might be discovered in the future. We observed eight effector sets in isolates of both collections, and multiple effector sets were detected in isolates from Copenhagen or London (Fig. 5c, d). The eight effector sets that are common to both collections consist of all 15 core effector genes, uniformly lack three accessory effector genes (*tle4b, tle2* and *tspE1a*) and vary between each other in the presence and absence of 20 accessory effector genes (Fig. 5e). The observed number of shared effector sets is expected by random chance (*P* value=0.634, Figs S18 and S19) and unlikely a result of geographic divergence. In sum, we found that the occurrence of an effector gene was similar across isolates of patients from two geographic regions.

## Discussion

We show strain-level variation in T6SS effector genes among clinical isolates of *P. aeruginosa* from people with CF. Whereas T6SS core effector genes are found among most isolates, the accessory effector genes are found in a fraction. By taking the patient origin of the isolates into account, we could show differences between individual patients in the colonizing clone types and sets of T6SS effector genes. Isolates that originate from the same individual and were collected on the same day of sampling differed in their effector set. Occurrence data on longitudinal isolates suggests rare events of effector gene loss during chronic infection of patients. Isolates from CF patients in Copenhagen and London show a similar occurrence of effector genes.

The observed variation in the occurrence of some T6SS effector genes likely results from the frequent gain and loss of these genes. Of note, we observed changes in the presence and absence of effector genes over very different scales in the phylogenies. Differences between clone types are indicative of such changes over long evolutionary scales during the diversification of the species, suggesting that diversification likely occurred pre-colonization. Differences between closely related isolates suggest very recent genomic deletions of either T6SS genes only or T6SS genes together with a large number of other genes. The isolates’ phylogeny and sampling timepoints suggest that some loss events likely occurred during chronic infection of the herein studied individuals. This seems especially likely for the loss of PA2066 and is slightly more speculative for the loss of *tse6*. Our findings demonstrate that changes in a strain’s T6SS effector gene, which are frequently made by genetic engineering in laboratory studies on reference strains, naturally occur among clinical isolates of people with CF and arise at low frequency during infection.

The fitness consequences of effector gene loss to *P. aeruginosa* during infection of the CF lung are unknown. Existing work focuses on the costs and benefits of the T6SS secretion apparatus *in vitro*. For example, an experimental evolution experiment by Taillefer *et al.* shows the loss of an active secretion apparatus in the absence of susceptible bacteria that can be killed [65]. This finding implies that T6SSs can be costly. A study of Perault *et al*. suggests that the loss of a functional secretion apparatus during infection of the lung results in reduced resistance to colonization by competing pathogens [19]. The herein observed loss of an effector gene together with an immunity gene would render bacteria susceptible towards an attack with the respective anti-prokaryotic effector. This observation differs from a scenario in which an orphan immunity gene remains and mediates protection in polymicrobial environments after the loss of the effector gene. The available data of the herein studied individuals did not enable us to test for positive, negative or neutral effects on bacterial fitness by effector gene loss in patients. Even if longitudinal data on bacterial populations of the patients would have been available, the loss of PA2066 together with multiple other genes would have made it impossible to conclude on the effects caused by the loss of this particular gene alone.

The differences in effector sets of T6SS effector and immunity genes between isolates sampled from the same patient raise the question of whether *P. aeruginosa* bacteria kill each other during infection. Experiments in the laboratory have shown T6SS-mediated killing between *P. aeruginosa* strains [15,16, 25, 55, 66,74]. Most of these observations are based on *P. aeruginosa* strains that (i) were genetically engineered to differ in an effector and immunity gene and (ii) were brought in contact during the experimental set-up. It was therefore unknown whether T6SS-mediated intraspecific killing is relevant to infection. We found naturally occurring variation in effector and immunity genes among clinical isolates of the same individual. Although it remains unclear whether these isolates are in direct contact during infection and end up killing each other, our findings make such a scenario more likely. Existing work in the laboratory demonstrated that contact-dependent bacterial killing by the T6SS rarely leads to a complete eradication of the susceptible strain but rather to a spatial segregation between strains with differing effector sets [75,76]. We therefore do not see a contradiction between putative T6SS-mediated killing between isolates in patients and the sampling of multiple isolates with differing effector sets in the same individual as observed in our study.

From the perspective of molecular pathogenesis, it is key to understand whether secretion systems with known anti-prokaryotic and anti-eukaryotic activity in the laboratory play a role in the infection of people with CF. Our data shows that the answer to this question is not always straightforward. In the assumption of a strong role of T6SS effectors in infection, one might expect (i) effectors facilitating colonization and infection in patients and therefore a higher number of effector genes in strong infectors, (ii) T6SS-mediated killing between isolates with differing effector sets and therefore all isolates of the same individual encoding the same effector set and (iii) selection against effector genes in patients and therefore frequent loss of multiple effector genes. Instead, we found (i) no association between the number of accessory effector genes and colonization and infection of people with CF, (ii) isolates with differing effector sets being isolated at the same date from the same individual and (iii) few effector genes being lost at low frequency in longitudinal samples. Although this data suggests a rather limited role of T6SS effectors in infection, further investigation will be needed. Core effector genes, which are present across strains, are less likely to contribute to strain-specific differences in colonization and infection as assessed here but might play a role in infection across strains. It is further possible that we have under-estimated the complexity of the effector gene repertoire as a result of missing effectors not found in reference strains. However, to reduce this possibility to a minimum, our identification of effector genes is not only based on reference strains but also on a comprehensive review of effector genes in clinical isolates of *P. aeruginosa* by Robinson *et al*. [34]. By restricting our analysis in this way, we ensured that the effector genes we analysed were validated and experimentally characterized. Further work is required on the identification of novel putative effector genes.

Taken together, we present the natural diversity of T6SS effector genes in clinical isolates across cohorts of people with CF. We found that the genetic diversity of effectors, which is routinely generated in the laboratory by genetic engineering of reference strains, does apply to clinical isolates that can naturally lose effector genes during infection. A key question for future work on a bigger cohort and higher sampling depth will be whether the phenotypes observed on laboratory strains with or without an effector gene equally apply to patients that are infected with strains with or without an effector gene.

## Supplementary material

10.1099/mgen.0.001555 Uncited Supplementary Material 1.

10.1099/mgen.0.001555 Uncited Supplementary Material 2.

## Abbreviations

BIC: Bayesian information criterion
CES: combined effector set
CF: cystic fibrosis
FDR: false discovery rate
NMDS: non-metric multidimensional scaling
NT: nucleotide
T6SS: type VI secretion system
