# Supplementary material for: Occurrence of type VI secretion system effector genes in longitudinal isolates of P. aeruginosa from people with cystic fibrosis
Source: Microb Genom. 2025 Nov 7;11(11):001555. doi: 10.1099/mgen.0.001555 (PMC12594252; doi:10.1099/mgen.0.001555)
Supplement: Uncited Supplementary Material 1. [file mgen-11-01555-s001.pdf]

Occurrence of type VI secretion system effector genes in longitudinal isolates of *P. aeruginosa* from people with cystic fibrosis

Supplementary Material

This file includes:

Figures S1-19

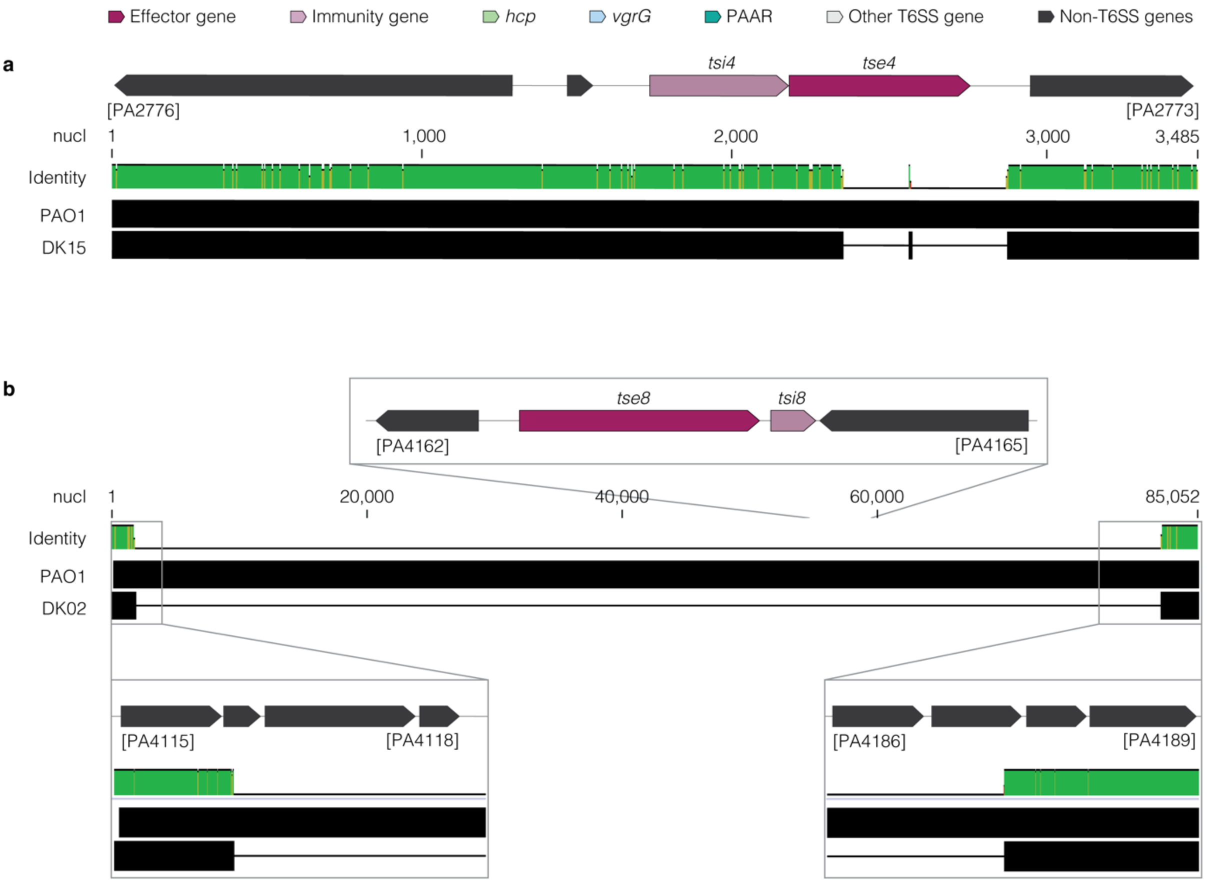

Figure S1. Two clone types lack H1-T6SS core effector genes *tse4* or *tse8*. a, Nucleotide alignment of PAO1 and isolate CPH_427 of clone type DK15 that does not encode effector gene *tse4* at full length. **b,** Nucleotide alignment of PAO1 and isolate CPH_165 of clone type DK02, which is missing a larger genomic region of ~80,000bp including H1-T6SS effector gene *tse8* and the cognate immunity gene *tsi8*.

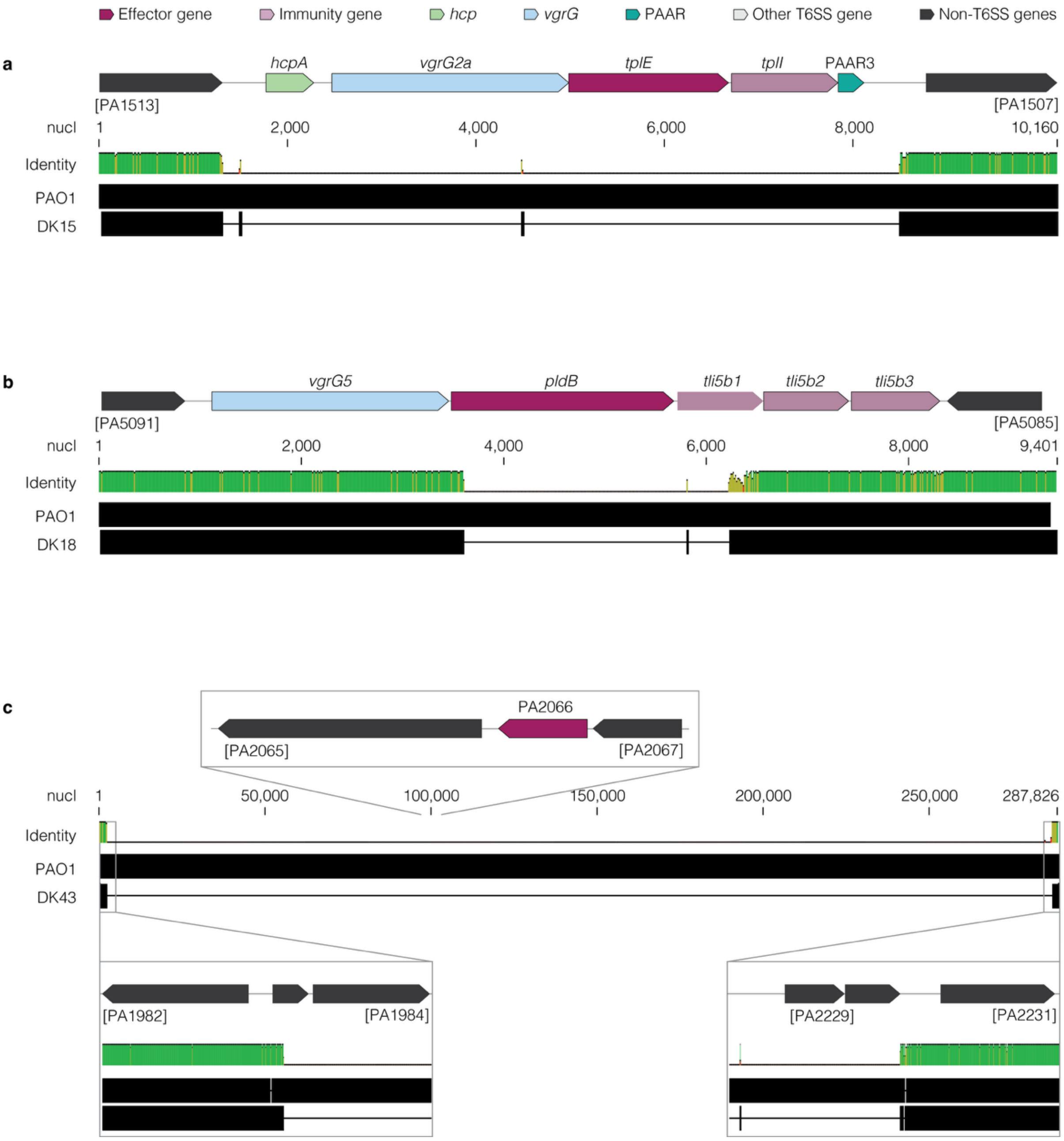

Figure S2. Three clone types do not encode H2-T6SS core effector genes *tplE*, *pldB*, PA2066. a, Nucleotide alignment of PAO1 and isolate CPH_427 of clone type DK15. The isolate is missing the effector gene *tplE* and neighbouring T6SS genes including the cognate immunity protein-encoding gene *tplI* and genes encoding for structural components of the T6SS apparatus (*hcpA*, *vgrG2a*, and PAAR3). **b**, Nucleotide alignment of PAO1 and isolate CPH_414 of clone type DK15. The isolate lacks most of the effector gene *pldB* and the corresponding immunity protein-encoding gene *tli5b1*. **c**, Nucleotide alignment of PAO1 and isolate CPH_373 of clone type DK43. CPH_373 lacks H2-T6SS core effector PA2066 as part of a big genomic deletion of ~285,000bp.

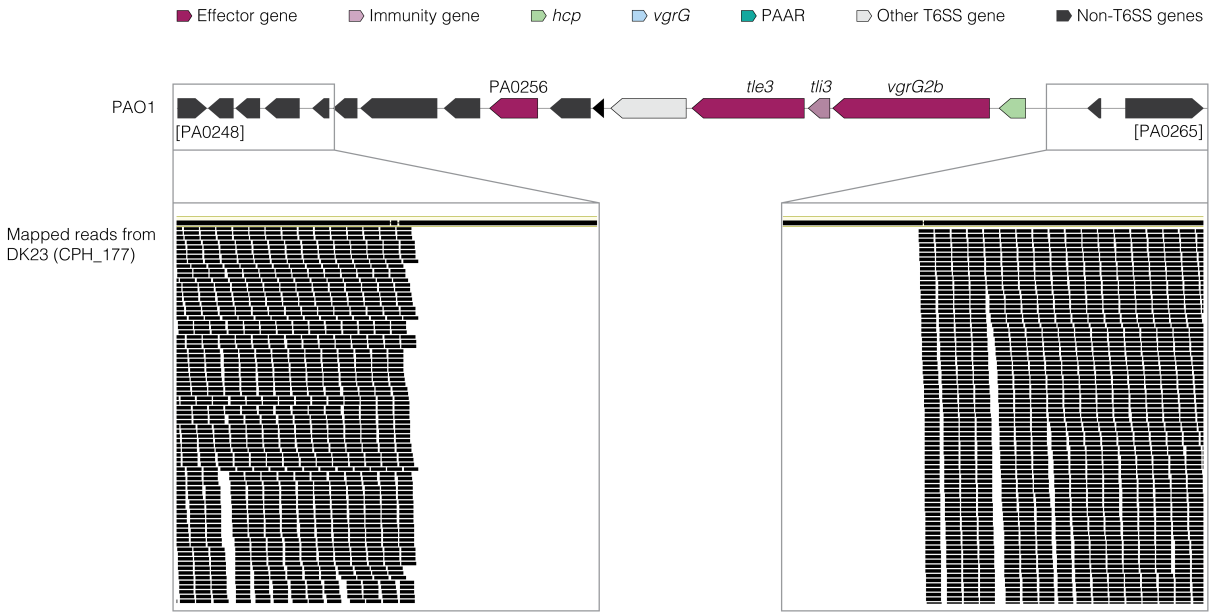

Figure S3. Isolates of clone type DK23 lack the H3-T6SS core effector gene PA0256. Reads of isolate CPH_177 (clone type DK23) were mapped to the reference strain PAO1. The inserts show the boundaries of the genomic region that is missing from the isolate.

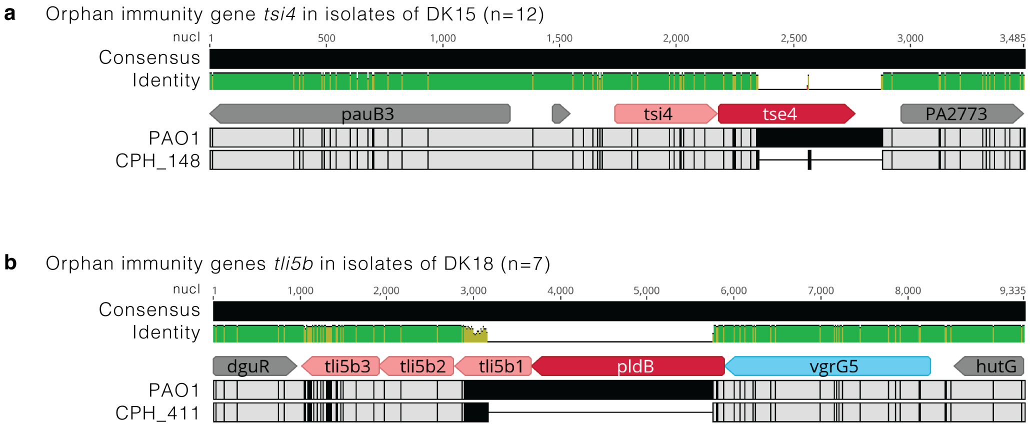

Figure S4. Examples of orphan immunity protein-encoding genes in the Copenhagen data set. **a**, Nucleotide alignment of PAO1 and CPH_148 indicating *tsi4* as an orphan immunity protein-encoding gene in CPH_148. Eleven other isolates (CPH_134, CPH_ 135, CPH_138_1, CPH_139, CPH_145, CPH_148, CPH_423, CPH_427, CPH_436, CPH_439_1, CPH_LRJ16, CPH_LRJ17) also have *tsi4* as an orphan immunity gene. **b**, Nucleotide alignment of PAO1 and CPH_411. Immunity protein-encoding genes *tli5b3* and *tli5b2* are found as orphan immunity-protein encoding genes in CPH_411 (here shown as an example) and six other isolates (CPH_412, CPH_412_1, CPH_413, CPH_413_1, CPH_414, CPH_440). Effector-encoding genes (dark red), immunity protein-encoding genes (light red), *vgrG* genes (blue), and neighbouring genes (grey) are coloured.

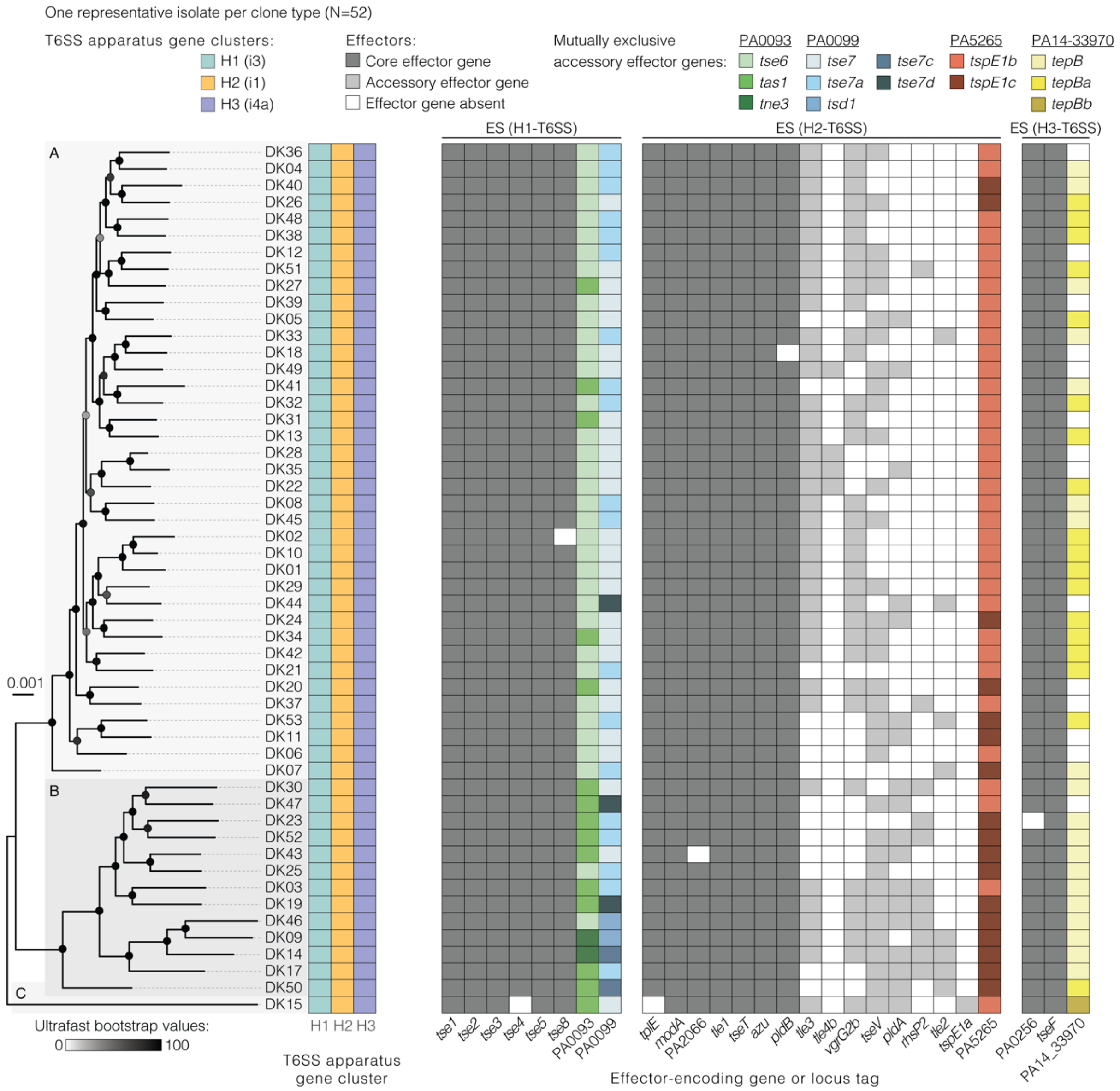

Figure S5. Phylogenetic tree of the clone types (n=52) based on a core genome alignment. For each clone type, the isolate with the earliest isolation date was chosen as a representative isolate for this analysis (Table S21). Boxes indicate the presence (filled box) or absence (white box) of T6SS apparatus gene clusters and effector genes. The T6SS subtype of the respective system is indicated in brackets. The maximum-likelihood tree was inferred with the GTR+F+R3 model and is midpoint rooted. Distances are indicated in substitutions per site. A tree with exact bootstrap values is shown in Figure S6.

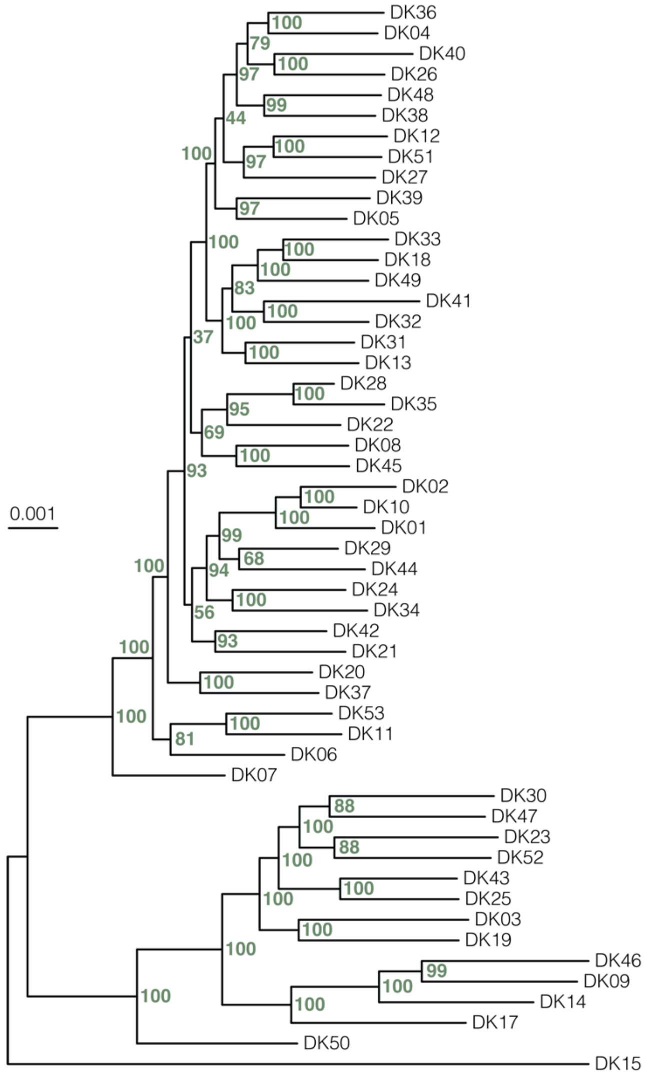

Figure S6. Maximum-likelihood phylogenetic tree from Figure 2b indicating exact ultrafast bootstrap values.

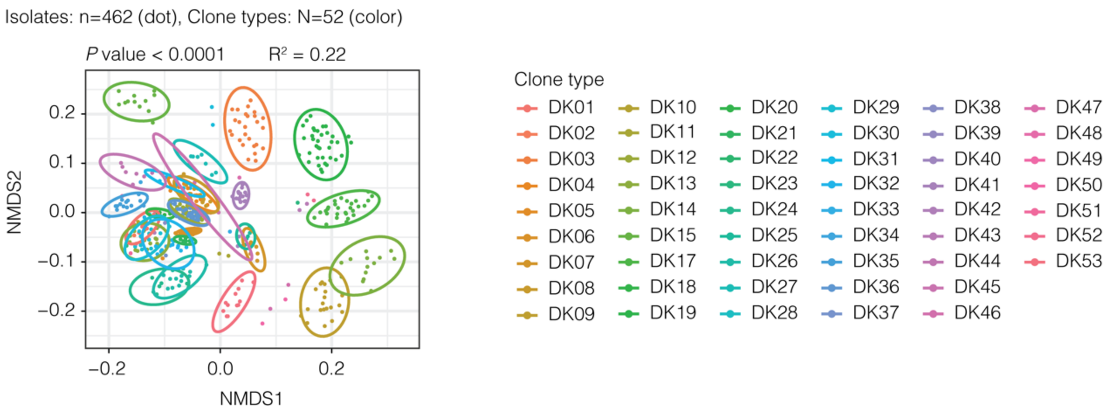

Figure S7. Extended NMDS plot from Figure 1e including a legend for the coloured dots.

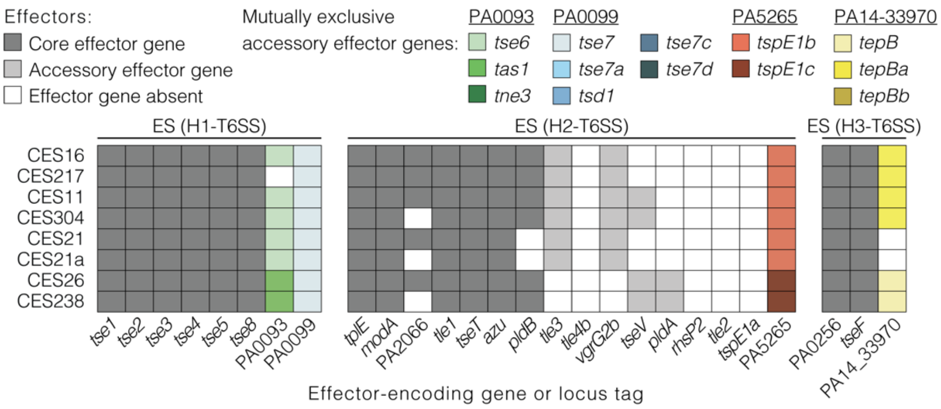

Figure S8. Graphical depictions of the CESs mentioned in Figure 1g. Each box indicates the presence (filled box) or absence (white box) of an effector gene. Dark grey boxes indicate core effector genes, light grey boxes accessory effector genes, and coloured boxes mutually exclusive accessory effector genes.

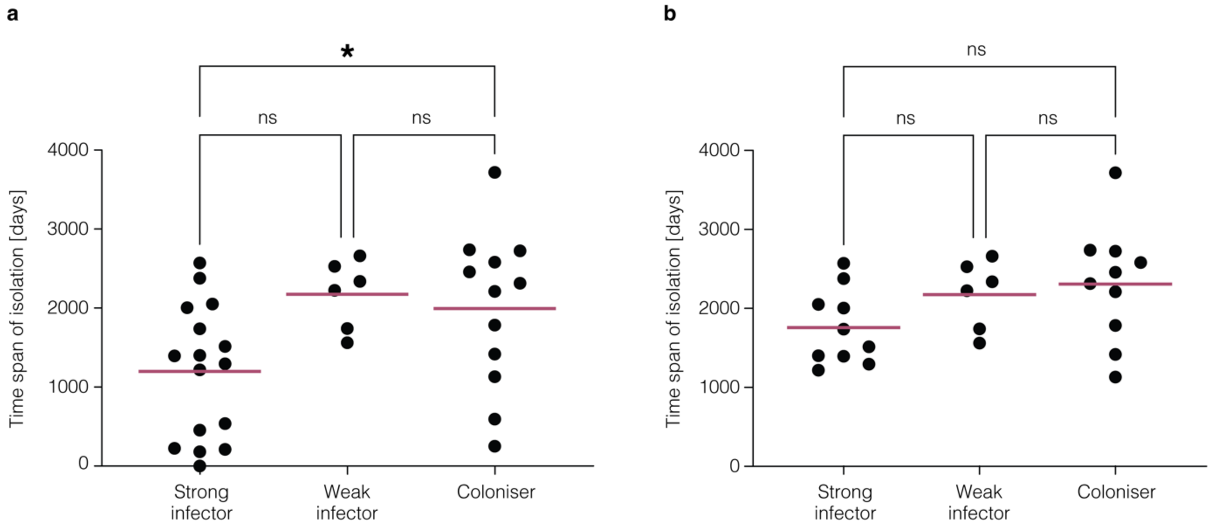

Figure S9. Dot plot indicating the time span of sampling of strong, moderate and weak colonisers before (**a**) and after (**b**) excluding individuals that had been sampled for less than 1000 days. Each dot indicates one clone type in the respective group. The red lines indicate the means. Statistical significance was tested using the Kruskal-Wallis test followed by a Dunn's multiple comparisons test. *, adjusted *P* value=0.05; ns, not significant.

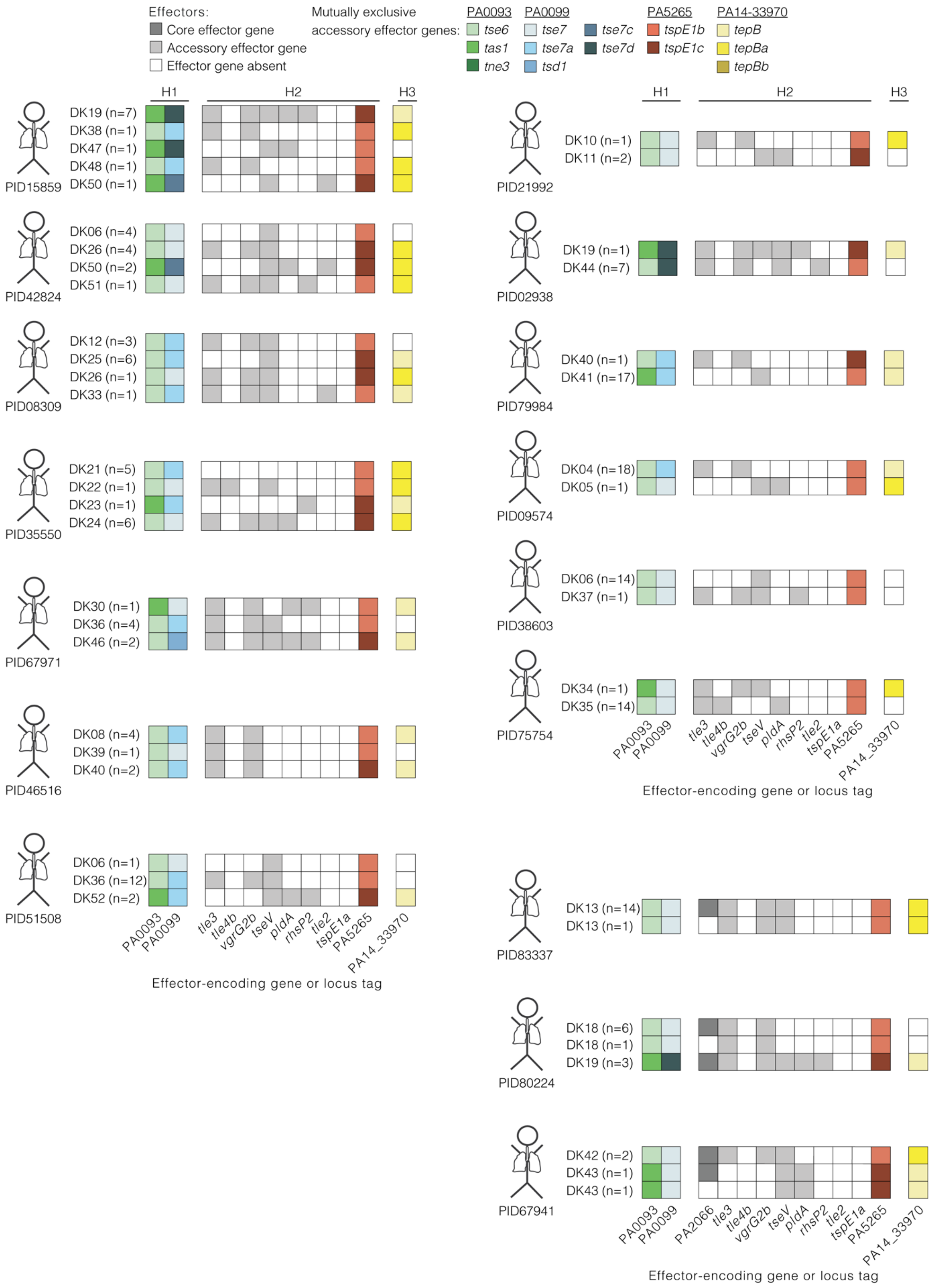

Figure S10. Isolates that were collected from the same individual at different time points differ in their T6SS effector sets. Graphical depictions of the different accessory effector sets that an individual with cystic fibrosis is exposed to. The number of isolates of a respective

clone type and effector set is indicated in brackets. Each box indicates the absence (filled in white) or presence (filled with grey or any of the indicated colours) of an effector gene. Only accessory effectors are shown, except for cases in which core effector genes differ in presence and absence between isolates of the same clone type.

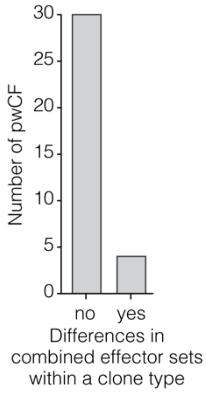

Figure S11. Most of the individuals (n=30) are colonised by multiple closely related isolates of the same clone type that share the same sets of T6SS effectors. Few individuals (n=4) are colonised by closely related isolates that differ in their presence and absence of T6SS genes.

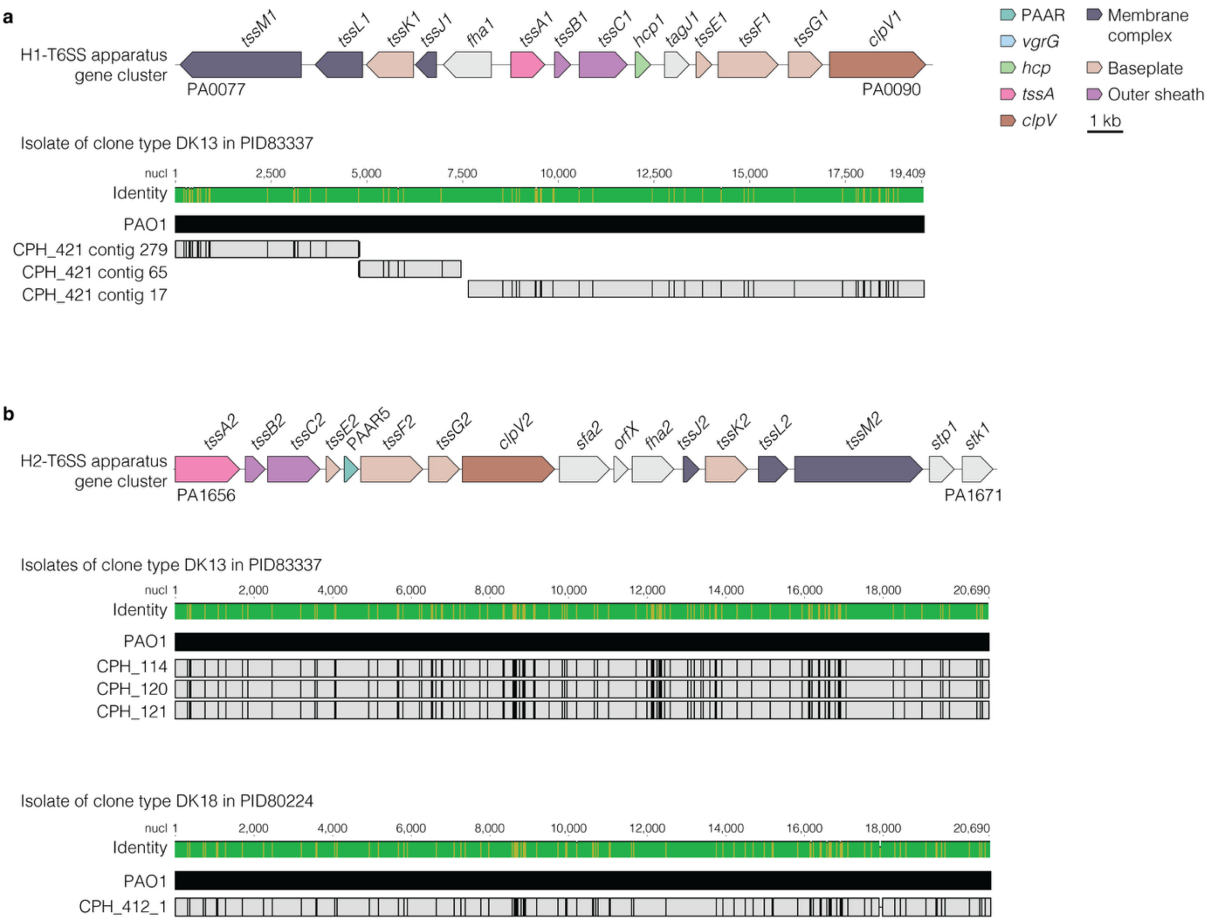

Figure S12. No indication for a dysfunctional T6SS in isolates that lost an effector gene. **a**, Nucleotide alignment of the H1-T6SS apparatus gene cluster of isolate CPH_421 to PAO1. Genes of the H1-T6SS belong to three separate contigs in CPH_421. **b**, Nucleotide alignment of indicated isolates of DK13 or DK18 and PAO1. Differences to the reference (PAO1) are indicated in black. These genomic differences do not lead to frameshift mutations or premature stop codons.

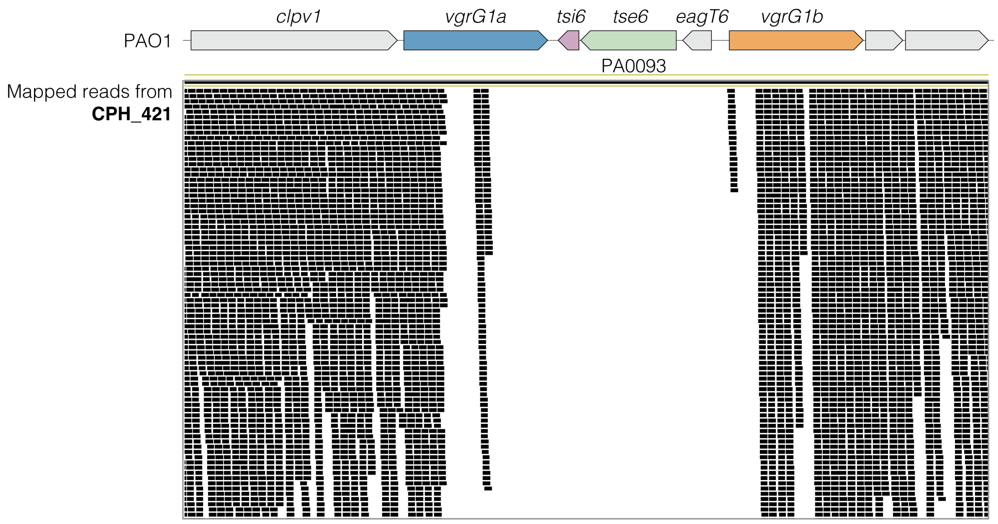

Figure S13. Isolate CPH_421 of clone type DK01 lacks the H1-T6SS effector gene *tse6* and neighbouring genes. Mapped reads from CPH_421 against the indicated genomic region of the PAO1 genome.

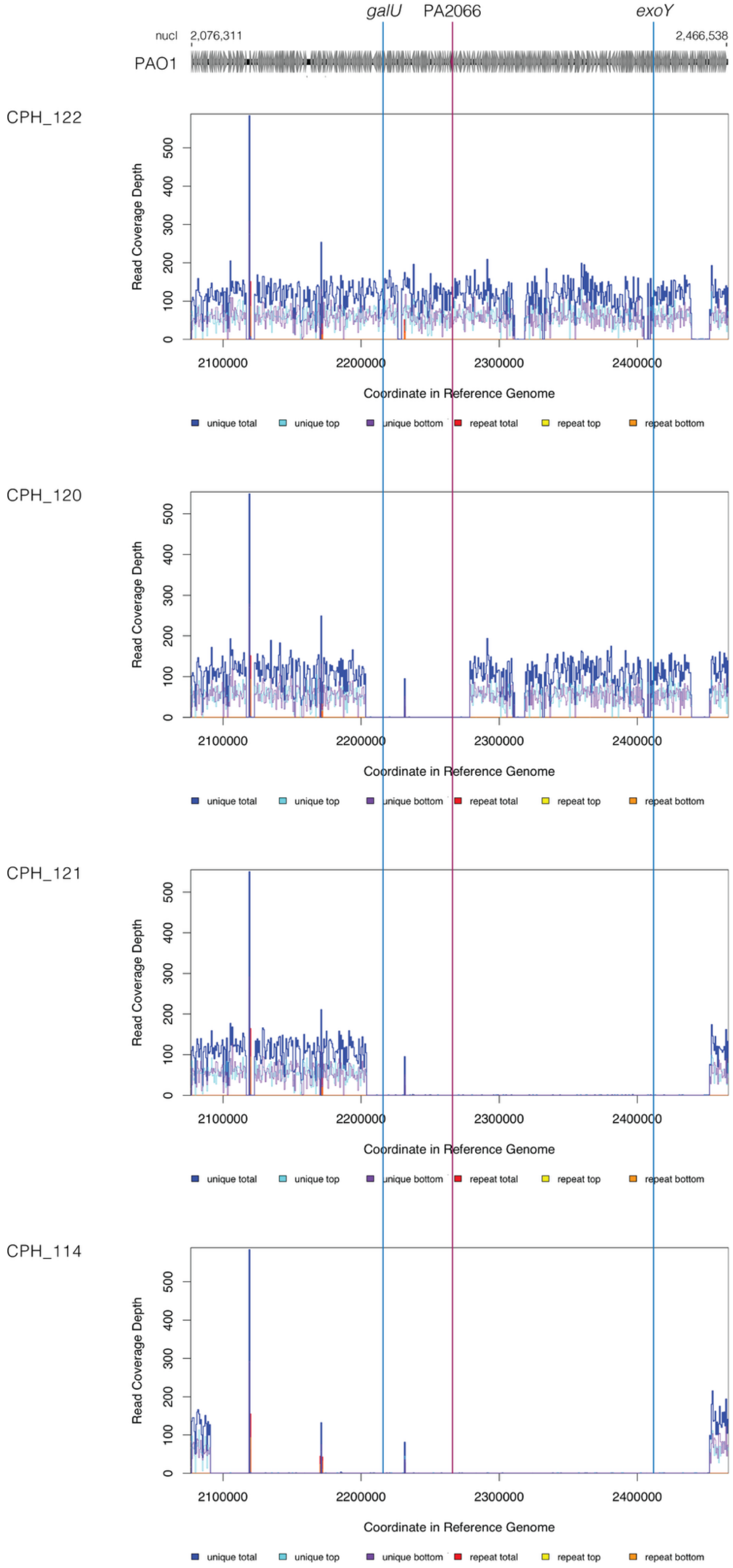

galU
PA2066
exoY
nucl
2,076,311
2,466,538
PAO1
CPH_122
CPH_120
CPH_121
CPH_114
Read Coverage Depth
0
100
200
300
400
500
2100000
2200000
2300000
2400000
Coordinate in Reference Genome
unique total
unique top
unique bottom
repeat total
repeat top
repeat bottom

86

Figure S14. The effector gene PA2066 is lost as part of a bigger genomic region in isolates from clone type DK13. Mapping of DK13 reads across the genomic region of PAO1 that includes PA2066. The plots were generated using breseq. PAO1 was used as a reference genome. PA2066, *galU* and *exoY* are indicated.

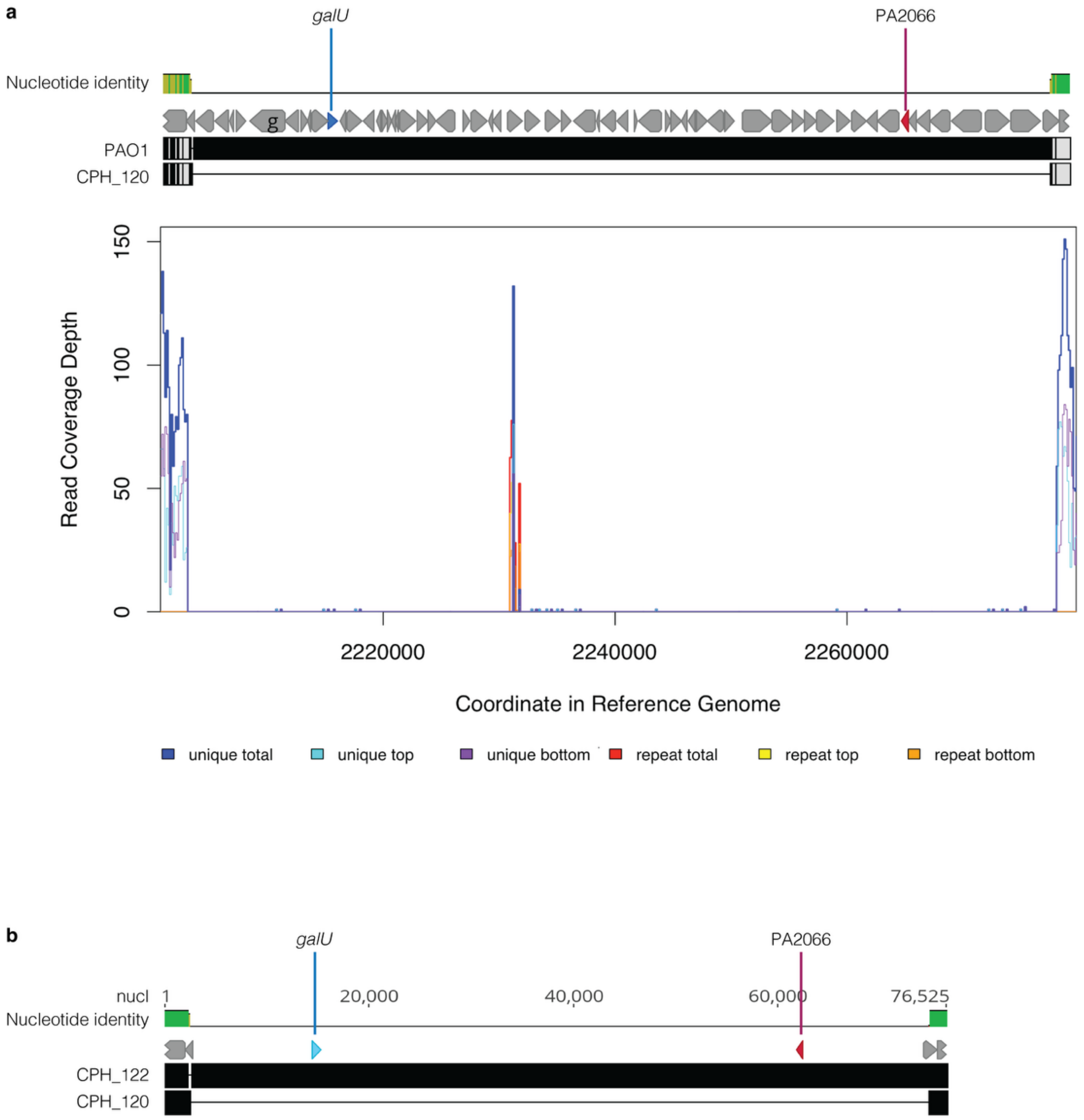

Figure S15. Effector gene PA2066 is missing as part of a bigger genomic deletion in isolate CPH_120. a, Nucleotide alignment of PAO1 and the assembled contig of CPH_120 of the same genomic region as for the plot on the read coverage depth (generated with breseq). **b**, Nucleotide alignment of CPH_122 (first isolate of patient PID83337) and CPH_120 (isolate lacking PA2066). Indicated are the effector gene PA2066 (red), the gene *galU* (blue), and genes present in both isolates (grey).

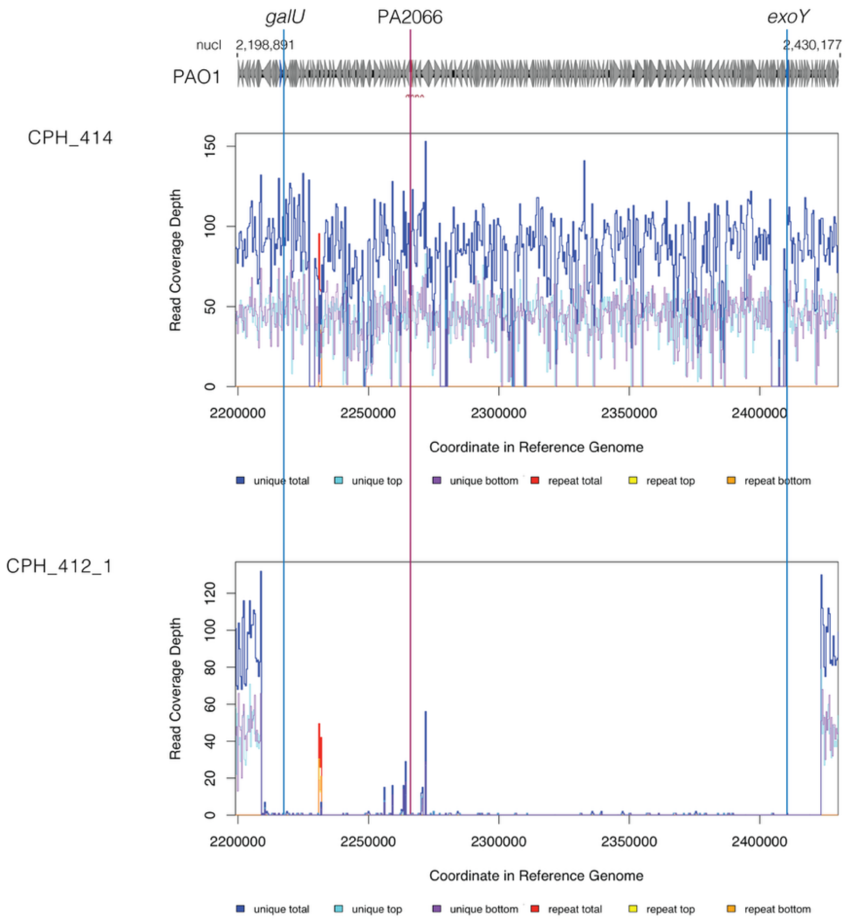

Figure S16. The effector gene PA2066 is absent as part of a bigger genomic region in isolate CPH_412_1 of clone type DK18. Coverage with DK18 reads across the genomic region including PA2066. Plots were generated using breseq. PAO1 was used as a reference genome. PA2066, *galU* and *exoY* are indicated.

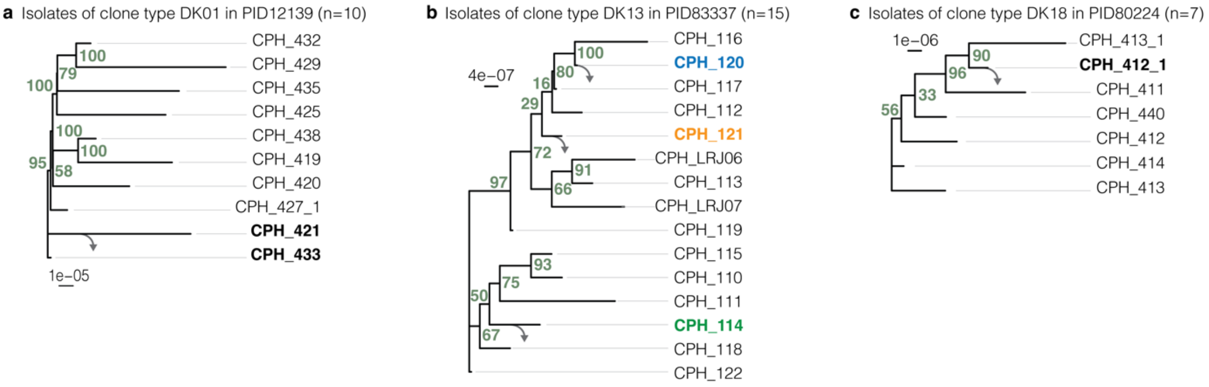

Figure S17. Phylogenetic trees from Figure 4 with indicated ultrafast bootstrap values.

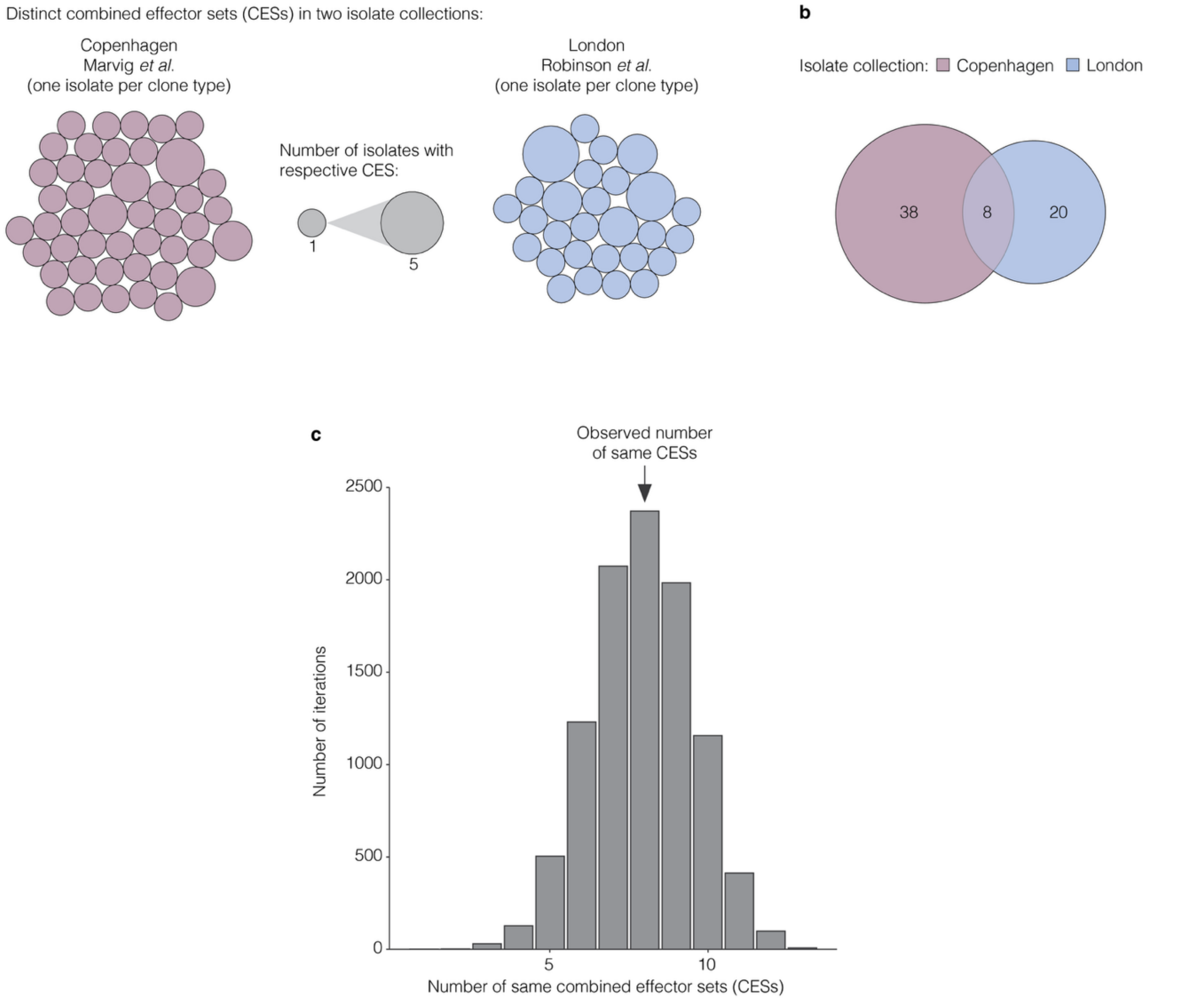

Figure S18. Effector sets in the two isolate collections after correcting for phylogeny. Only one effector set is considered per clone type. **a**, Bubble graph indicating the distributions of distinct combined effector sets (CESs) in a subset of isolates from Copenhagen (one isolate per clone type, n=52) and isolates from London (n=36). Each bubble indicates one distinct CES. The size of the bubble indicates the number of isolates with the respective CES. **b**, Venn diagram indicating unique and shared CESs of the isolate collections. **c**, Bar graph indicating the number of iterations over the number of same effector sets after randomly splitting the two isolate collections into two groups (number of total iterations 10,000).

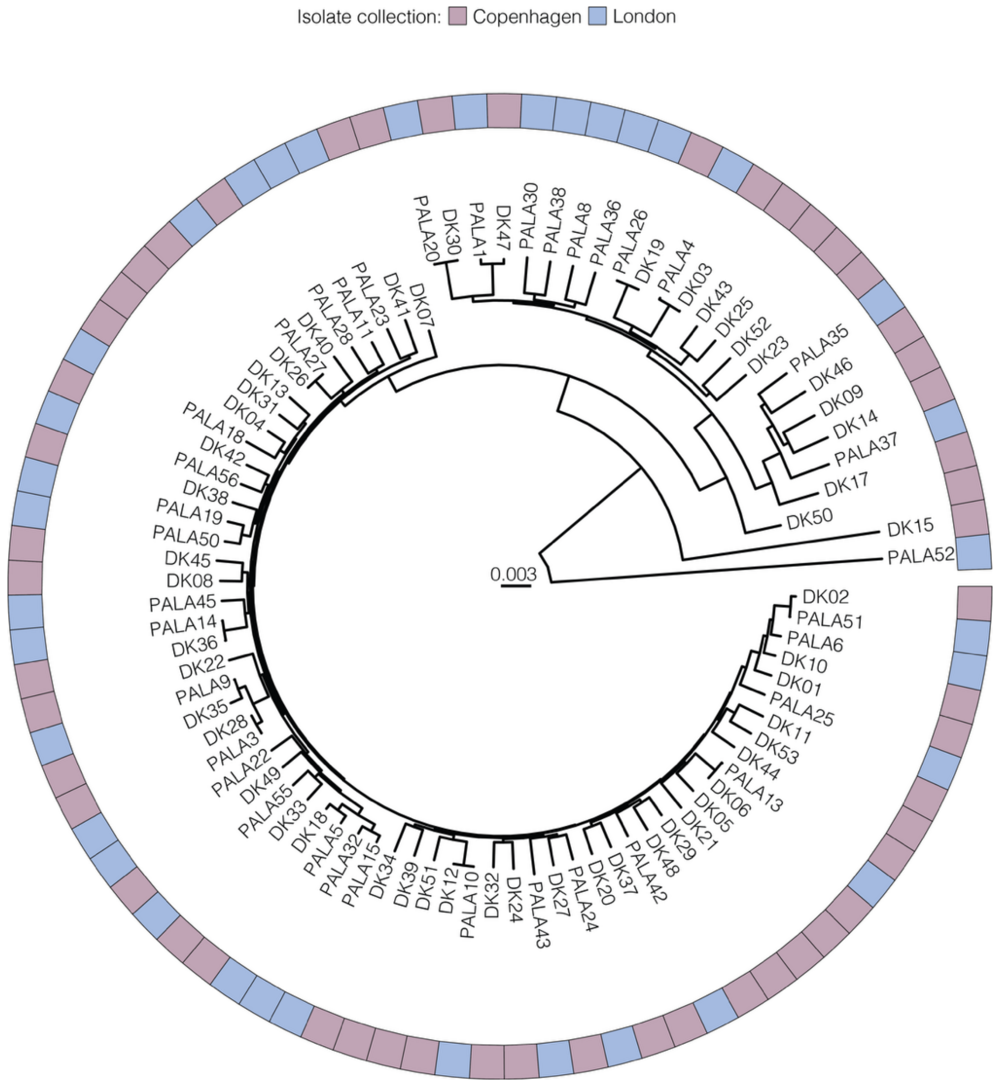

Figure S19. Maximum-likelihood phylogenetic tree of one isolate per clone type of the Copenhagen isolate collection (n=52, see Table S22 for sequence IDs) and the isolate collection from London (n=36). The coloured boxes in the outer ring indicative the isolate collection. The tree is based on a core genome alignment and computed using the GTR+F+R3 model. The tree is midpoint rooted and distances are shown in substitutions per site.
